# The role of pragmatic mechanisms in referential communication and categorization: An emergent communication model

**DOI:** 10.1371/journal.pcbi.1014326

**Published:** 2026-05-26

**Authors:** Kristina Kobrock, Xenia Ohmer, Elia Bruni, Nicole Gotzner

**Affiliations:** Institute of Cognitive Science, Osnabrück University, ‌‌Osnabrück,‌‌ Germany; Interdisciplinary Transformation University IT:U, AUSTRIA

## Abstract

We model pragmatic mechanisms of referential communication and categorization in a multi-agent framework of emergent communication. Pragmatic theories and experimental work predict that speakers consider the context in their choice of referring expressions. In addition to this context-based reasoning, utility-based pragmatic reasoning about the listener’s likely interpretation of an utterance influences the speaker’s production choices. We aim to investigate these two factors and their role in referring expression generation and categorization in a computational model of language emergence and language use. We model communication in interaction and consider an efficiency tradeoff between speaker and listener utilities. Our results show that an emerging language becomes more effective, ambiguous, and efficient when speakers and listeners communicate in a shared context. This is achieved by an efficient tradeoff between production and comprehension where languages can afford to be simpler in production when they are sufficiently informative in context, placing more burden on the listener’s side. We further demonstrate that incorporating utility-based pragmatics, as modeled with the Rational Speech Acts framework, improves the linguistic efficiency of language use only in languages that emerged with a shared context between interlocutors, but not in languages that emerged without such contextual information during training. We conclude that context-based pragmatics plays a role in referential communication and categorization by shaping an emerging language. Efficient reference in a communicative situation can benefit especially from utility-based pragmatics if the language that is being used has emerged in context. This might suggest that utility-based pragmatics hinges on mechanisms that naturally emerge when context is available during the evolution of a language. In summary, we show that human-like language and category systems emerge as an optimized tradeoff between speaker and listener needs in interaction, i.e., under efficiency considerations of simplicity and informativeness.

## Author summary

This study investigates pragmatic mechanisms of referential communication and categorization in a multi-agent framework. We study how speakers and listeners interact to create an efficient language, balancing the need for clear communication with the cost of producing messages. Our results show that an emerging language becomes more effective, ambiguous, and efficient when speakers and listeners communicate in a shared context. This mechanism of context-based pragmatics plays a crucial role in shaping the language, allowing speakers to be simpler in production when listeners can infer the correct meaning in context. Additionally, we discovered that utility-based pragmatics modeled with the Rational Speech Acts framework impacts language efficiency, particularly when the language has emerged in context. Our findings suggest that utility-based pragmatics is closely tied to the mechanisms that naturally emerge when context is available during language evolution. The resulting language and category systems resemble those of human languages. This research contributes to our understanding of how efficient communication systems emerge in social interactions, and based on communicative need.

## Introduction

The languages we speak influence which categories we know and talk about. The idea that language shapes cognition in this way has become popular as the (weak) Sapir-Whorf hypothesis, or linguistic relativity hypothesis [1,2]. One instance of the claim that language shapes cognition is that conversation shapes category structure [3]. A famous example is the popularized claim from Boas [4] and Sapir [1] that Inuit have more words for subcategories of snow than languages spoken in warmer climates. While early works frequently used the term ‘Eskimo’, we use ‘Inuit’ here because ‘Eskimo’ is largely dispreferred by the native populations of Alaska due to its colonial heritage [5]. The popularized claim about words for snow has been recently revisited in a large-scale analysis of linguistic and meteorological data [6]. The authors found that languages that use the same linguistic form for both snow and ice tend to be spoken in warmer countries. They relate this finding to the lower communicative need to talk about snow and ice in these regions [6]. This is in line with the efficient communication hypothesis which predicts that human languages are optimized for efficient communication, a claim that has gained traction in many subfields of linguistics such as language evolution [7,8], pragmatics [9–12] and more general proposals [6,13–17].

Following the efficient communication hypothesis, the main idea for how language shapes category structure is that languages have words for things that are relevant, i.e., where there is a communicative need [6,18]. This can be exemplified by the following relationship [6]:


$$\begin{array}{c} \text{Environment}\to \text{Communicative Need}\to \text{CategorySystem} \end{array}$$ (1)

If there is a communicative need to talk about subcategories in an environment, then the category system will be more fine-grained than if there is no such need [6]. More fine-grained categories are more informative than broader categories, but they are also more complex and require more storage space in memory. It has been proposed that, due to cognitive constraints, both languages and category systems tend to find an optimal tradeoff between simplicity and informativeness [6,8,16–20].

How this tradeoff is optimized can be better understood when considering interactive communication. Let’s assume that a lexical system evolves in a simple conversational setup in which a speaker describes an object to a listener. If this system was optimized for the speaker, then in an extreme case, the system would only include one word that can be used to describe all objects. This optimizes simplicity and keeps memory storage for the speaker to a minimum. A system that is optimized for the listener, on the other hand, includes one label for each object. This optimizes informativity of the lexicon and it makes it easy for the listener to identify the correct target when hearing a word. This principle was proposed as the Principle of Least Effort by Zipf [17] and the idea has been also taken up later [16,21]. Relatedly, Rosch et al. [19,20] have proposed a similar principle for category structure: The Principle of Cognitive Economy states that a category system should provide maximum information with the least cognitive effort. An optimal category system would thus optimize a tradeoff between having many categories with very fine-grained distinctions between them and having fewer categories with coarser distinctions between them. A category system with many categories is optimized for informativity: Many properties of one category can be predicted by knowing only one property, e.g., the label. A category system with few categories is optimized for cognitive economy because fewer categories need to be learned and stored in memory [19,20]. This tradeoff can also be thought of as a tradeoff between speaker and listener needs in a communicative situation, specifically in a referential communicative situation. Barr and Kronmüller have argued that referential communication is especially critical for categorization, specifically category learning and category use [3]. Following this, we use referential communication to study categorization in a communicative setting where speakers and listeners interact. This allows us to study communicative and categorization efficiency as a tradeoff between speaker and listener needs.

The question that we tackle in this paper is the following: Can the efficient communication hypothesis predict category structure in a language? Or, more specifically, which pragmatic mechanisms allow for an optimization of the tradeoff between simplicity and informativeness and lead to efficient communication about categories on a conceptual hierarchy? Evidence for the efficient communication hypothesis has been found in the context of kinship [18] and color [22–25] systems. However, this hypothesis has not yet been tested for more general categories or even for the most ubiquitous category system that we use: How we categorize the things around us in hierarchical structures that are also called levels of abstraction [19], e.g., DALMATIANS, DOGS and ANIMALS. To study the role of categorization in shaping communicative needs and, ultimately, how categories are being referred to in a language, we look at the referential communication of categories at different levels of abstraction and use a modeling approach with controlled symbolic datasets that include hierarchical categories. Crucially, we consider the role of pragmatic mechanisms in shaping communicative needs in the process of language evolution through repeated interactions because pragmatic principles guide communicative interactions.

One central puzzle for theories of efficient communication is how to account for ambiguity and redundancy observed in human language [16,26,27]. Ambiguity in human languages has been related to pragmatic considerations [16,28,29]. Pragmatic theories assume that language is interpreted in context and in a referential situation where speakers use an underinformative or ambiguous expression, context usually helps to disambiguate the intended meaning [16,27]. Context-based pragmatics can thus reconcile ambiguity with the efficient communication hypothesis because speaker and listener share the workload: The listener disambiguates ambiguous messages based on contextual considerations such that the speaker can use shorter, more efficient and ambiguous utterances. But, humans have also been shown to frequently overspecify referring expressions with atypical or salient properties such as color even in situations where these properties are not needed to unambiguously identify a target referent in a given context. On first sight, this is at odds with the efficient communication hypothesis and Gricean maxims of conversation, especially the quantity maxim which states that speakers should provide as much information as needed and not more [30]. But recent proposals have shown that the redundant expressions provide information that helps listeners to identify the target referent, e.g., by mentioning atypical features or providing salient information in complex scenes that facilitate visual search [9,10,26,31,32]. This can be explained by the idea that speakers take into account the listener’s needs and want to guide the listener toward identifying the correct referent as quickly as possible [26,32]. Reasoning about the listener’s likely interpretation is a classic case of utility-based pragmatics and it can be straightforwardly modeled with the Rational-Speech-Act framework [26,33,34]. Utility-based pragmatics can thus explain how redundancy can be considered efficient and reconcile redundancy with the efficient communication hypothesis.

The main objective of this work is to find out how categorization influences an emerging language and what role language and pragmatics play in this process. The goals of our study are thus twofold: First, we investigate the influence of our manipulations of different pragmatic mechanisms on the (use of the) emerging language. We ask whether efficient communication, formalized as a tradeoff between speaker and listener needs, emerges from interaction and pragmatic considerations, when communicating concepts at different levels of abstraction. We use an emergent communication paradigm to model the joint emergence of categories and category labels in an interactive communicative framework that takes into account speaker and listener needs. Second, we draw inferences from the emerged linguistic system to the category system and analyze how concepts at different levels of abstraction are communicated in the language. To this end, we use the trained emergent communication models and test them in specific communicative situations and under consideration of different pragmatic mechanisms, such as context-based and utility-based pragmatics.

### Modeling approach

**Fig 1:**
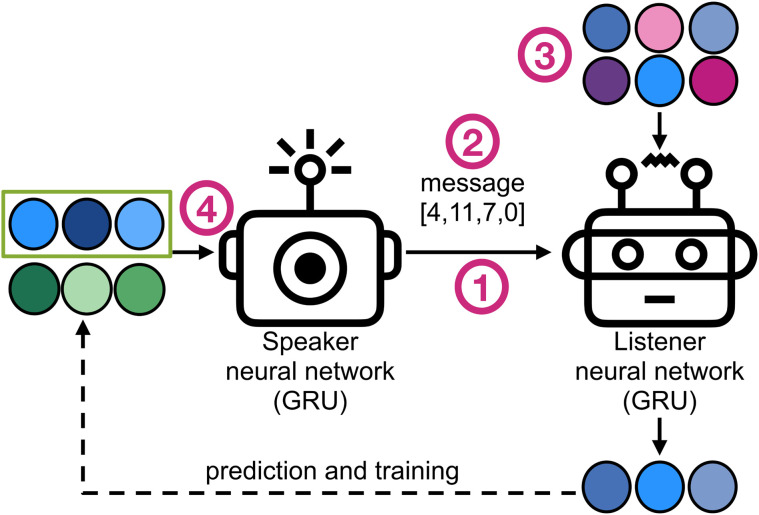
Model architecture. In this example, the speaker has to communicate the concept of a blue circle and the listener has to identify all blue circles. The loss function rewards shorter messages from the speaker (efficiency) and correct selections from the listener (informativeness). This figure was created by the first author.

The overarching goal of this research is to model context-based and utility-based pragmatic mechanisms in a communicative interaction that involves categorization, and to find out under which circumstances they become especially useful and make the (use of the) emerging language particularly efficient. Our research makes use of an emergent communication model [35–38]. The architecture of the model is visualized in Fig 1 and implements four communicative principles that are outlined below.

First, the model is interactive, i.e., it takes into account and models the fact that communication happens in interaction between a speaker and a listener agent to model the tradeoff between efficiency and informativeness. On the speaker side, efficiency is achieved by a length cost pressure that incentivizes speakers to use shorter messages. On the listener side, informativeness is achieved by training the agents on communicative success. To meet these requirements, our model should have at least a basic one-way interaction between a speaker who sends a message and a listener who receives a message. The model that we use utilizes such a basic interaction which is also utilized in standard linguistic theoretical and experimental frameworks [33,39].

Second, the model should make as little prior assumptions about communication as possible. Therefore we do not use a model of language use on an existing language, for example English, but rather let a proto-language emerge in interaction between artificial agents. The goal is to observe what a language might look like given the constraints we implement. If in our agent-based models we observe that a language emerges that shares certain characteristics with human language, we can conclude that these characteristics can emerge even in simple neural-network-based models that implement some central characteristics of communication, such as a reference-based interaction, but do not implement other uniquely human constraints, such as language universals or a particular grammar [40].

Third, the model takes into account that communication should be grounded. For grounded communication, it is crucial that communication is situated in a world - this can be a toy world to keep the complexity of the model small. This is done by simplifying communication to referring. The agents need to solve a reference game where the speaker has to describe a target and the listener has to select the target from a set of distractors. The emerging language is a meaningful mapping between messages and objects of the world that are being referred to. This simple task and setting have an additional advantage: We have full control over the environment, i.e., we can construct the properties of the objects and thereby control for every parameter of the model: The objects that should be referred to and the context in which the reference takes place. In other words, when controlling for the targets and the context, we can control for how much information needs to be communicated to achieve successful communication. In our datasets, objects are comprised of symbolic attributes which can take a certain number of values. Our notion of informativeness is defined by the number of attributes that are being communicated, where more attributes mean more information. The amount of information that needs to be communicated relates closely to efficiency.

Fourth, the model should allow us to investigate categories at different levels of abstraction. To achieve this goal, we implement a concept-level reference game that the agents need to solve [37,41,42]. The concept-level reference game is similar to a simple reference game. The only difference is that instead of a single target object, a target concept needs to be communicated. This target concept is constructed by combining several target objects which share certain attributes. The more attributes are shared between objects belonging to the same target concept, the more specific, or subordinate, the concept, and the less attributes are shared between target objects, the more generic, or superordinate, the concept. For example, it has been shown in experimental work that when speakers observe two targets, a PARROT and a DOG, they use the superordinate referring expression “animal” to refer to both [42]. Relating to the notion of informativeness introduced above, the more generic the concept, the less information is conveyed by its label and the more specific the concept, the more information is conveyed by its label. The concept-level reference game ensures that the agents in our model communicate about concepts at different levels of abstraction instead of lower-level features that are not part of the concept hierarchy.

Finally, to answer our research question on the role of context-based and utility-based pragmatic mechanisms for categorization, we need to implement these different kinds of pragmatic mechanisms. We implement both conditions in two experiments with minimal assumptions and the goal is to observe whether the mechanisms will emerge in the model given the constraints we place. To test context-based pragmatics in Experiment 1, all we add is that speakers will be able to observe a context. Note that contrary to the speaker, the listener always observes a context to ensure that their task, to select the correct target concept, is non-trivial. There is no additional utility function or incentive for the agents to make use of the context. We will evaluate whether our manipulation, i.e., whether speakers can observe the context, leads to languages emerging with different characteristics. This means that we will model and investigate the influence of the possibility of context-based pragmatic reasoning (on the speaker side) on the evolution of a language. Our main hypothesis in Experiment 1 is:

1. If context is available and shared between the speaker and the listener, then the emerging language will be more efficient than if context is not available.
  To test for utility-based pragmatics in Experiment 2, we will implement speakers which are modeled with the Rational Speech Act (RSA) framework. We will use the trained models and languages from Experiment 1 and evaluate whether our manipulation in Experiment 2, i.e., whether speakers engage in level one pragmatic reasoning about the listener’s likely interpretation of a message, influences the use of an already emerged language in a specific communicative situation. Here, our main objective is to see how the use of a language is influenced by the reasoning of the speakers. Our model provides the means to investigate under which circumstances an emergent language can be used efficiently by speakers in a communicative situation. Our main hypothesis in Experiment 2 is:
2. Speakers will use more effective and efficient messages if they reason about the utility of their utterance for the listener than when they do not.
  We describe the complete methodology including further details on the implementation in the Methods section at the end of this paper.

### Theoretical and empirical background

Pragmatic considerations regarding referring expressions are very well studied experimentally in humans. We know of a number of interacting factors that influence a speaker’s choice of referring expression. First, the target that the speaker wishes to communicate restricts the set of possible utterances to those referring expressions which actually communicate (properties of) the target. For example, if a speaker wants to describe a dog running across the street, they could reasonably choose one of the following expressions: $U_{descriptive}=\{``dog",``dalmatian",``animal"\}$. In contrast, choosing $U_{non-descriptive}=\{``cat",``vehicle",``thought"\}$ have a very low probability of being chosen by an informative speaker. Second, the context in which a target is embedded and, more generally, the context of the communicative act, play a role in the production of referring expressions. For example, even though both the expressions “dalmatian” and “dog” sufficiently describe the target object, speakers are more likely to choose “dog” than “dalmatian”. This is due to the basic-level effect, i.e., a preference for basic-level referring expressions in production [19,42,43]. The more informative expression “dalmatian”, however, is preferred in a context where a dalmatian needs to be discriminated from a husky. In experimental pragmatics, it has been found that speakers are more likely to choose a more informative referring expression, i.e., a subordinate referring expression (“dalmatian”), or a modified nominal referring expression (“tall glass”), if an object of the same type is present in the context [42–45].

Pragmatics does not only play a role in specific communicative interactions, but also in the evolution and emergence of referring expressions. Such long-term effects over repeated interactions can be studied with experimental paradigms such as iterated learning and artificial language learning experiments, as well as in computational modeling paradigms such as the emergent communication paradigm. These paradigms stem from a long tradition of using interactive games to study communicative interactions and language evolution [46–48]. Focusing on experimental studies, artificial language learning experiments have been used to study how the context in which target objects need to be communicated, can explain properties of an emerging lexicon [8,39,49,50]. For example, it has been shown that abstract references, i.e., references that can be used to refer to more than one object, emerge in coarse contexts [39,50] and that contextual predictability shapes signal autonomy [8]. Focusing on computational modeling, the iterated learning computational framework and game theoretic approaches were very influential [46–48]. Notably, especially the research tradition following Steel’s Talking Heads experiments focuses on the joint evolution of conceptualizations, i.e., mappings between perception and meanings, and lexicalizations, i.e., mappings between meanings and words, in the communicative interaction between autonomous agents [47,51,52]. Our approach is based on the integration of Deep Neural Networks and Multi-agent Reinforcement Learning to study the interactions of neural-network-based agent which allows for improved scalability and flexibility [35,53–55].

The emergent communication framework is well-suited to model the role of pragmatics in communication [36,41,56,57]. Previous work uses RSA agents, i.e., agents which reason about a messages’ utility based on their interlocutor’s likely interpretation in a way predicted by the RSA framework, to show what the availability of utility-based pragmatic reasoning adds to referential communication [56,57]. For example, Fang et al. show how the redundant use of color adjectives can be explained by a systematic variation of the environments in which the neural network agents have been trained in a communication task [57].

Classically, the RSA framework is used to computationally model speakers’ and listeners’ behavior in communicative tasks such as the reference game [26,33,34,58]. While the framework predicts human production and comprehension data well, it is debated whether speakers and listeners engage in recursive pragmatic reasoning to formulate and interpret messages (all the time) [59]. This relates to the communicative efficiency hypothesis presented above. If ambiguity in language is considered desirable from an efficiency perspective, then this is because the context is usually informative and allows for a rapid disambiguation of ambiguous utterances in context [16]. However, research in experimental pragmatics has shown that inferences on the listener side are not necessarily cheap (as suggested for example by Horn as the division of pragmatic labor [60]), but rather that deriving meaning can be computationally and cognitively costly [21,61]. The question under which circumstances processing of ambiguous utterances is slow or fast is an ongoing discussion and an interesting research topic from the perspective of communicative efficiency [62]. In our paradigm, we can compare context-based (supposedly fast) with utility-based (supposedly slow) pragmatic reasoning [63] and compare the efficiency of productions based on contextual vs. utility-based pragmatic reasoning and the effectiveness in terms of communicative success. One central criticism of the RSA modeling paradigm is that it presupposes a small fixed set of possible messages and that interlocutors only reason about this small space of possible messages in a given context. This is cognitively implausible and quickly becomes intractable [56,64]. Reasoning about a small space of messages thus might be a good approximation for specific referential situations, but can never capture the full inventiveness and flexibility of human language. Modeling pragmatic agents based on neural network architectures can help to scale the applicability of the RSA framework [56,65–67]. In our model, we use all messages that have been generated during training for the RSA computation. Instead of the computationally costly Bayesian inference in the classical RSA paradigm, neural-network-based approaches for RSA-based referring expression generation approximate the inference, for example by directly taking into account the interpretation of an internal listener model [56,57,65–67]. This means that we exploit the fact that for the comparatively small emerging proto-languages and neural-network-based approaches, the computation of RSA inferences becomes computationally tractable.

### Overview of experiments

**Fig 2:**
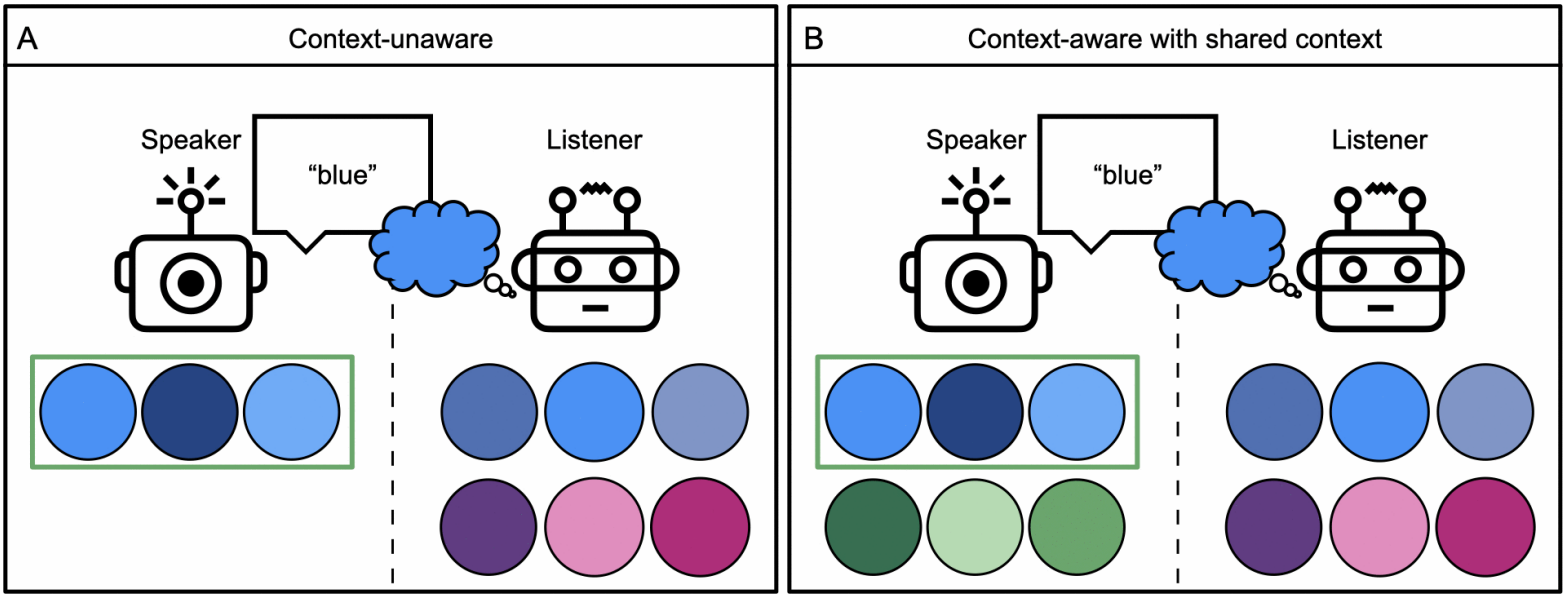
Game scenarios in Experiment 1. A: Context-unaware - The speaker agent only observes the set of target objects, i.e., the target concept (displayed in a green frame for visualization purposes). B: Context-aware - The speaker agent observes the target concept in a context, i.e., a set of target objects (visualized in blue) and a set of distractor objects (visualized in green). This figure was created by the first author.

In Experiment 1, we study how the availability of context influences an emerging language in a concept-level reference game, i.e., during the referential communication of target concepts. Target concepts consist of multiple target objects to ensure referential communication on a conceptual level opposed to communication about lower-level and irrelevant features. We compare two game scenarios: In the baseline scenario, a speaker agent communicates a concept to a listener agent which has to select all target objects that satisfy the concept (see Fig 2A). In our baseline, the speaker does not have access to the context. We refer to these agents as being trained “context-unaware”. Only the listener is presented with a context to make the task nontrivial.

In this baseline scenario, cooperative and informative speakers have to communicate all attributes relevant to a concept, i.e., all *fixed* attributes, because they do not know in which context the concept is presented. We compare this baseline scenario to a scenario that implements a shared context between the speaker and the listener agent. We call this scenario the “context-aware” scenario (see Fig 2B). It differs from the context-aware condition presented in previous works [37,68,69] by assuming a shared context between the interlocutors. Specifically, in previous work, while the context condition was shared between speaker and listener, it was not excluded that the distractor objects that formed the context differed from the target concept in different attributes. This means that it was possible that the speaker observed small blue triangles as targets in a context of small red triangles, whereas the listener was presented with small blue triangles in a context of large blue triangles. In the model we present here, however, the attribute(s) in which the distractor objects differ from the target concept are shared between speaker and listener. This makes the context more informative which should lead to a more efficient use of the context. The amount of shared context between two interlocutors is thought to influence communication in pragmatics theories and has been called mutual cognitive environment [70] or common ground [71]. By adding this type of context, our model can model all three types of context that are thought to be involved in referential communication, the immediate situational context, the shared context between interlocutors and the historical discourse context [8]. The neural-network agents do not have an explicit memory for past discourse, but the historical discourse context influences the emerging language indirectly by influencing the neural network weights during training. We hypothesize that in the context-aware condition, speakers and listeners may develop a language that is ambiguous and relies on context to disambiguate meanings. That means that speakers may communicate fewer than all fixed attributes and that this should not impede listener performance.

**Fig 3:**
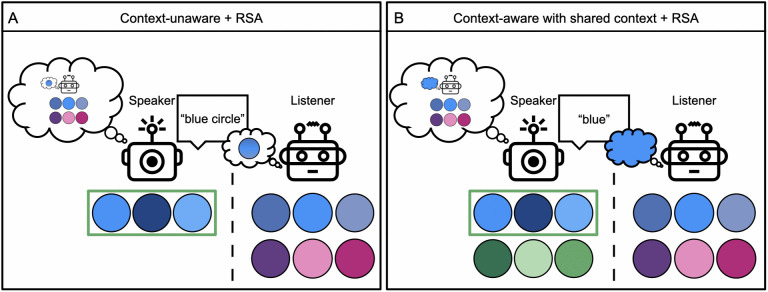
Game scenarios added in Experiment 2. A: Context-unaware + RSA - Speaker agents trained in the context-unaware condition reason about the listener’s likely interpretation of their message. B: Context-aware + RSA - Speaker agents trained in the context-aware condition reason about the listener’s likely interpretation of their message. This figure was created by the first author.

In Experiment 2, we study the use of the languages that emerged in the first experiment during inference, i.e., after training of the models. We compare four different conditions: First, we gather agent interactions on the test dataset from the context-unaware and the context-aware agents that have been trained in Experiment 1. Second, we equip both the context-unaware and the context-aware speaker agent with utility-based pragmatics implemented following the Rational-Speech-Act (RSA) model (see Fig 3A–3B). Note that the implementation of the RSA speaker does not differ between conditions. What differs between the context-aware + RSA and the context-unaware + RSA condition are the language and the internal listener that were shaped during training in the context-aware or context-unaware condition, respectively, in Experiment 1. This means that the RSA reasoning mechanism is the same in both conditions and that any differences we observe are due to the way in which the RSA speakers are able to leverage and make use of the languages that emerged with or without context. We will compare how well agents that have been trained context-unaware or context-aware perform on the test dataset and assess what the RSA implementation changes with regard to the baseline performance. We also investigate the properties of the messages that are used in these settings. We hypothesize that RSA speaker agents should come up with efficient and highly informative messages because they are able to reason about the selection the listener is likely to make based on the message they send and because RSA implements a cost term that penalizes inefficient communication. For a better overview, we summarize the predictions and measures in Table 1. Further information on the measures and metrics used to analyze the results can be found in the subsection Evaluation in the Methods section at the end of this paper. All source code for the modeling and statistical analyses can be found under the following link: https://github.com/kristinakobrock/pragmatic-mechanisms (DOI: https://doi.org/10.5281/zenodo.15497186).

**Table 1:** Summary of predictions and measures.

Experiment	Conditions	Prediction	Measures
Experiment 1: Emergence (Training)	• Context-unaware • Context-aware	• More ambiguity and efficiency in context-aware compared to context-unaware condition	• message length • entropy scores
Experiment 2: Use (Inference)	• Context-unaware • Context-unaware+RSA • Context-aware • Context-aware+RSA	• More efficient language use the more pragmatic information becomes available	• message length • entropy scores • frequency ranks • qualitative analysis

## Results

In tables and figures, we report means and standard deviations over five runs with random initialization. For the statistical analyses, we used R version 4.4.1 [72] and the R-packages brms [73] and bayestestR [74]. We fit Bayesian hierarchical models with sum-contrast-coded population-level (fixed) effects for condition and group-level (random) effects for datasets with uniform priors. The models were run for 2000 iterations with a warm-up period of 1000 iterations. All Rhat values were <1.01 and all effective sample sizes (ESS) were >1000. More details can be found in S2 Appendix. All reported effects are certainly existing, determined by the Bayesian test of probability of direction (pd), i.e., *pd* > 99.9% and significant, determined by the Bayesian test for Region of practical equivalence (ROPE), i.e., < 1% in ROPE, except denoted otherwise.

### Experiment 1: Context-based pragmatics

To assess the role of context in referring to concepts at different levels of abstraction, we compared the baseline game scenario in which the speaker has no access to the context (context-unaware condition) with a game scenario in which the speaker has access to a context that is shared between the two interlocutors (context-aware condition with shared context). Note that the context-unaware scenario by definition cannot have a shared context because speakers are not presented with any distractor objects but make their decision of which message to communicate solely based on the target objects.

First, we looked at the performance, i.e., accuracies observed on the training, validation and test datasets for both conditions. Table 2 shows that while we observed high performance in both settings, context-aware agents generally showed higher performance than context-unaware agents, i.e., validation accuracies are ≥0.96 in the context-aware and ≥0.86 in the context-unaware condition. We observed a main effect for condition (M = -0.03, CrI=[-0.04, -0.02], pd = 100%, ROPE=[-0.01, 0.01], 0% in ROPE) in a Bayesian hierarchical model predicting validation accuracy by condition. When comparing the different types of accuracies within a condition, we saw that context-unaware training is more prone to overfitting (see training trajectory plots in S1 Fig), i.e., showed a larger difference between training and validation accuracies, than context-aware training. We report the number of epochs needed to train the different conditions with early stopping in S1 Table.

**Table 2:** Mean accuracies on the training, validation and test datasets.

	Context-unaware			Context-aware shared context		
**Datasets**	**train**	**validation**	**test**	**train**	**validation**	**test**
(3,4)	0.97 ± 0.01	0.92 ± 0.01	0.79 ± 0.02	0.99 ± 0.01	0.96 ± 0.01	0.93 ± 0.02
(3,8)	0.98 ± 0.00	0.95 ± 0.01	0.87 ± 0.03	0.99 ± 0.01	0.98 ± 0.01	0.97 ± 0.01
(3,16)	0.96 ± 0.02	0.95 ± 0.04	0.94 ± 0.04	0.98 ± 0.02	0.97 ± 0.04	0.96 ± 0.05
(4,4)	0.96 ± 0.02	0.91 ± 0.02	0.88 ± 0.02	0.99 ± 0.00	0.98 ± 0.01	0.98 ± 0.01
(4,8)	0.93 ± 0.04	0.90 ± 0.06	0.90 ± 0.06	0.98 ± 0.02	0.98 ± 0.02	0.98 ± 0.02
(5,4)	0.91 ± 0.07	0.86 ± 0.10	0.85 ± 0.09	0.99 ± 0.01	0.98 ± 0.01	0.98 ± 0.01

Mean accuracies and standard deviations over five runs are calculated on the train, validation and test data splits for each dataset and both conditions.

**Fig 4:**
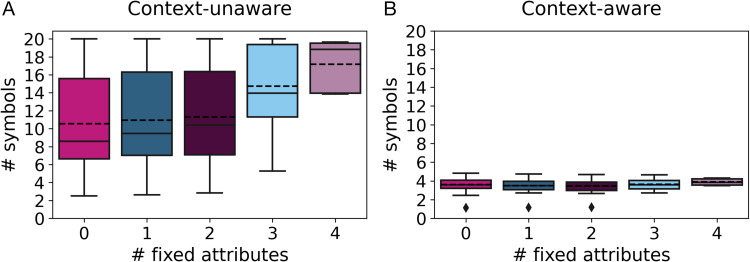
Message lengths per concept hierarchy level. Concept specificity, or the amount of information that needs to be communicated, increases with the number of fixed attributes that is shared among target objects from more generic concepts (with fewer fixed attributes) on the left, to more specific concepts (with more fixed attributes) on the right.

Second, we looked at the message-level efficiency of the emerging language. While the effective vocabulary size, i.e., the number of symbols from the vocabulary that have actually been used by the agents, was maximal in both conditions, we can see differences in message lengths. Fig 4A–4B shows the lengths of messages referring to concepts at a certain hierarchy level. The concept hierarchy is displayed as number of fixed attributes on the x-axis. The higher the number of fixed attributes, the more specific is a concept. Fig 4A–4B shows that the messages that emerged in the context-unaware condition tended to be at least six symbols longer than the messages that emerged in the context-aware condition with shared context. In the context-unaware condition, we observed a trend that the more information needed to be communicated about a concept, i.e., the higher the number of fixed attributes, the longer the messages. This was supported by a main effect for condition (M = 3.18 CrI=[2.49, 3.92], pd = 100%, ROPE=[-0.57, 0.57], 0% in ROPE) and an interaction effect between condition and number of fixed attributes (M = 0.64, CrI=[0.26, 1.02], pd = 99.90%, ROPE=[-0.57, 0.57], 35.37% in ROPE) in a Bayesian hierarchical model predicting message length by condition. This effect is certainly existing but of undecided significance judging the ROPE criterion.

Third, we calculated entropy-based scores on the emerging message-concept mapping. Specifically, we compute *Normalized Mutual Information* (NMI) as a measure of one-to-one mappings between messages and concepts, *effectiveness*, as a measure of one-to-many mappings between messages and concepts, and *consistency*, as a measure of many-to-one mappings between messages and concepts. More detailed descriptions and mathematical notations can be found in the Methods section under Evaluation. Table 3 shows that the NMI, the effectiveness and the consistency score were higher in the context-unaware condition than in the context-aware condition. We fit three Bayesian hierarchical models predicting the respective score (NMI, effectiveness, consistency) by the condition. We found a main effect for condition in all three models, i.e., predicting NMI (M = 0.14, CrI=[0.13, 0.16], pd = 100%, ROPE=[-0.02, 0.02], 0% in ROPE), predicting effectiveness (M = 0.22, CrI=[0.20, 0.25], pd = 100%, ROPE=[-0.02, 0.02], 0% in ROPE), and predicting consistency (M = 0.04, CrI=[0.03, 0.05], pd = 100%, ROPE=[-0.01, 0.01], 0% in ROPE). This means that the amount of one-to-one mappings between messages and concepts was lower, and that the emerging language contained more many-to-one and one-to-many mappings between messages and concepts, in the context-aware condition compared to the context-unaware baseline.

**Fig 5:**
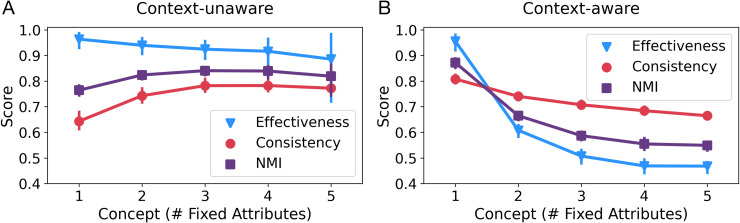
NMI, consistency and effectiveness scores for each level of the conceptual hierarchy.

How is it that the context-aware agents had such an ambiguous language but still achieved very high performance? We looked at entropy-based scores calculated for each level of the conceptual hierarchy to address this question. Fig 5A–5B shows the information-theoretic scores comparing the context-unaware to the context-aware condition. The effectiveness score is closely related to communicative success, i.e., how likely the listener is to select the correct target objects. If effectiveness is high, this means that meaning uncertainty is low. Effectiveness is generally higher in the context-unaware than in the context-aware condition as shown in Table 3 and in Fig 5A–5B. Interestingly, in both context-aware and context-unaware conditions, generic concepts with only one fixed attribute were referred to almost with maximal effectiveness. Thus, there was no meaning uncertainty for messages that were used to refer to generic concepts, i.e., they effectively singled out the targets. The effectiveness score had the tendency to drop the more specific a concept was which means that there were more one-to-many mappings between messages and concepts and meaning uncertainty the more specific a concept. While in the context-unaware condition the effectiveness score dropped gradually and had a linear trend, in the context-aware condition, the effectiveness score dropped exponentially with each level of the conceptual hierarchy. The consistency score can be viewed as a measure for of the speaker’s tendency to select the same message for a given target concept in repeated iterations. If the consistency score is high, this means that there is low signal uncertainty. In other words, the speaker’s choice of message is straightforward and easy. In the context-unaware condition, we observed higher consistency, i.e., lower signal uncertainty, the more specific the concepts. In the context-aware condition, we observed lower consistency, i.e., higher signal uncertainty, the more specific the concepts. The NMI curves of both conditions reflected the described trends: In the context-unaware condition, the NMI score was more closely oriented to the consistency score, i.e., there were more one-to-one mappings the more specific a concept. In the context-aware condition, NMI followed the effectiveness curve and decreased with increasing number of fixed attributes, suggesting that there were fewer one-to-one mappings between messages and concepts with specific than with generic concepts. The above described trends were supported by three hierarchical Bayesian models predicting the respective scores by the interaction of condition and concept hierarchy level, i.e., fixed attributes. We found a main effect for condition and concept hierarchy level, as well as an interaction effect for these two predictors in models predicting the NMI, effectiveness and consistency (see Table C in S2 Appendix).

**Table 3:** Mean entropy-based scores, i.e., NMI, effectiveness and consistency.

	Context-unaware			Context-aware shared context		
**Datasets**	**NMI**	**effectiveness**	**consistency**	**NMI**	**effectiveness**	**consistency**
(3,4)	0.81 ± 0.01	0.90 ± 0.05	0.74 ± 0.01	0.59 ± 0.03	0.52 ± 0.04	0.68 ± 0.01
(3,8)	0.87 ± 0.00	0.99 ± 0.00	0.77 ± 0.00	0.58 ± 0.01	0.47 ± 0.02	0.75 ± 0.00
(3,16)	0.86 ± 0.04	0.83 ± 0.10	0.90 ± 0.07	0.59 ± 0.09	0.48 ± 0.11	0.77 ± 0.01
(4,4)	0.85 ± 0.00	0.97 ± 0.02	0.77 ± 0.01	0.54 ± 0.03	0.44 ± 0.04	0.69 ± 0.01
(4,8)	0.86 ± 0.02	0.89 ± 0.05	0.84 ± 0.05	0.55 ± 0.06	0.45 ± 0.07	0.73 ± 0.01
(5,4)	0.83 ± 0.08	0.88 ± 0.16	0.79 ± 0.01	0.51 ± 0.02	0.41 ± 0.02	0.69 ± 0.01

Mean entropy scores and standard deviations over five runs are calculated for each dataset and both conditions.

## Discussion

In summary, we observed that a more ambiguous and efficient language emerged in the context-aware condition than in in the context-unaware condition which is in line with our predictions. This finding was supported by measures of communicative success (accuracy), of efficiency (message length), and of ambiguity (entropy-based scores). We found that communicative success on the validation dataset was substantially higher in the context-aware than in the context-unaware condition. Messages were substantially shorter in the context-aware than in the context-unaware condition. Together with the high performance, this means that the languages that emerged in the context-aware condition are likely simpler which makes them well interpretable and well suited for generalization purposes. When interpreting the entropy-based scores we obtained for each language, it was evident that the languages that emerged in the context-aware condition were more ambiguous. We found fewer one-to-one mappings between messages and concepts shown by the substantially lower NMI score in the context-aware compared to the context-unaware condition. The substantially lower effectiveness scores in the context-aware condition mean that there is high meaning uncertainty and that listeners have to choose the most likely interpretation from more than one candidate interpretation. Together with the high accuracies, this suggests that listeners successfully make sense of the ambiguous messages they receive. In the context-unaware condition, effectiveness scores were quite high, suggesting that the messages that the listeners received were not ambiguous, and it is thus straightforward for the listeners to interpret the messages and identify the correct targets. The consistency scores suggested that there is a certain amount of signal uncertainty, or many-to-one mappings between messages and concepts, in the emerging languages of both conditions with little, but substantially, more many-to-one mappings in the context-aware than in the context-unaware condition.

The effectiveness and consistency scores can also provide insight into how the languages emerged as a tradeoff between speaker and listener needs. The efficient communication hypothesis predicts that an efficient language for the speaker will have as few distinct messages as possible that can be used in multiple contexts. This translates into a high consistency score, i.e., low meaning uncertainty. An efficient language for the listener, on the other hand, will have many distinct messages because that makes interpretation easier. This translates into a high effectiveness score, i.e., low meaning uncertainty. In the context-unaware condition, we observed higher effectiveness than consistency. This means that the emerging languages in this condition were more optimal for listeners than for speakers. The languages have messages that can be interpreted unambiguously which makes the listener’s task especially easy. In other words, the tradeoff between production and comprehension is balanced by putting burden on the speaker who has to select a message from a set of multiple possible utterances and send quite long messages that make target identification easy for the listeners. In the context-aware condition, we observed the opposite pattern, namely that consistency is higher than effectiveness. This puts more burden on the listener’s side who has to interpret the meaning of polysemous messages in context. The speaker, on the other hand, has a lower burden than the listener. This is due to the lexicon having evolved towards a simpler lexicon with one-to-many mappings between messages and concepts. This ambiguity makes the resulting language more efficient. This is only possible in the context-aware condition because the context is informative and can help the listener to resolve the ambiguity of the polysemous messages.

In the referential communication task we employ, agents have to communicate about concepts ranging from specific to generic presented in a context of varying granularity. Looking at how concepts of varying specificity are encoded in the emerging languages provides insights into the category structure learned by the agents. We have conducted a concept-level analysis of the entropy-based scores to assess this. In both conditions, we observed that generic concepts with only one fixed attributes are referred to with almost maximal effectiveness, i.e., very low meaning uncertainty. Moving towards more specific concepts, the effectiveness drops gradually in the context-unaware condition and exponentially in the context-aware condition. The exponential drop in the context-aware condition reflects the fact that the number of possible contexts, and thus the possibility to exploit these contexts for efficient communication, increases exponentially, the more attributes are fixed in a target concept. This trend can be also observed in natural languages where the same specific concept can be referred to with different labels, e.g., “animal”, “dog”, or “dalmatian”, depending on the context. The most generic concepts, on the other hand, e.g., ANIMAL or THING, tend to have only one label. We conclude that the availability of a shared context not only makes the emerging language more efficient, but also shapes the category system and how concepts at different levels of abstraction are referred to.

### Experiment 2: Utility-based pragmatic reasoning

The goal of the second experiment was to investigate the influence of utility-based reasoning on the situational reference to concepts at different levels of abstraction in different contexts. We looked at situational reference after a language has emerged in the context-unaware and context-aware conditions from the first experiment. This means that we investigated the interactions on a test dataset with concepts that have not been included in the train and validation datasets.

First, we compared accuracies collected on the test dataset for context-unaware agents, context-unaware trained agents with RSA, context-aware agents, and context-aware trained agents with RSA. Table 4 shows that test accuracies were higher in the context-aware than in the context-unaware condition. This means that agents generalized better in the context-aware condition. Regarding utility-based pragmatic reasoning, accuracies in the context-unaware condition did not improve with RSA but RSA seemed to worsen performance of context-unaware agents. In the context-aware condition, however, utility-based pragmatics lead to similar performance. These effects were supported by a Bayesian hierarchical model predicting test accuracies by the interaction of context-based (context-unaware vs. context-aware) and utility-based (without RSA vs. with RSA) pragmatics. We found a main effect for context-based pragmatics (M = -0.08, CrI=[-0.09, -0.07], pd = 100%, ROPE=[-0.01, 0.01], 0% in ROPE), a main effect for utility-based pragmatics (M = 0.03, CrI=[0.02, 0.04], pd = 100%, ROPE=[-0.01, 0.01], 0% in ROPE) and an interaction effect (M = 0.03, CrI=[0.02, 0.04], pd = 100%, ROPE=[-0.01, 0.01], 0% in ROPE). Following a reviewer’s suggestion and to better contextualize these results, we estimated the amount of ambiguous games in our dataset where we would in principle expect level-1 RSA reasoning to help the speaker to select the best utterance. These games make up about two thirds of the games in our test datasets (see S3 Appendix).

**Table 4:** Accuracies on the test dataset.

Dataset	Context-unaware	+ RSA	Context-aware	+ RSA
(3,4)	0.79 ± 0.02	0.87 ± 0.04	0.93 ± 0.02	0.96 ± 0.02
(3,8)	0.87 ± 0.03	0.82 ± 0.03	0.97 ± 0.01	0.99 ± 0.01
(3,16)	0.95 ± 0.04	0.72 ± 0.03	0.96 ± 0.07	0.95 ± 0.09
(4,4)	0.88 ± 0.02	0.75 ± 0.04	0.98 ± 0.01	0.98 ± 0.01
(4,8)	0.89 ± 0.07	0.66 ± 0.02	0.97 ± 0.02	0.96 ± 0.04
(5,4)	0.88 ± 0.09	0.67 ± 0.07	0.99 ± 0.01	0.98 ± 0.03

Mean accuracies and standard deviations over five runs are calculated for each dataset.

**Fig 6:**
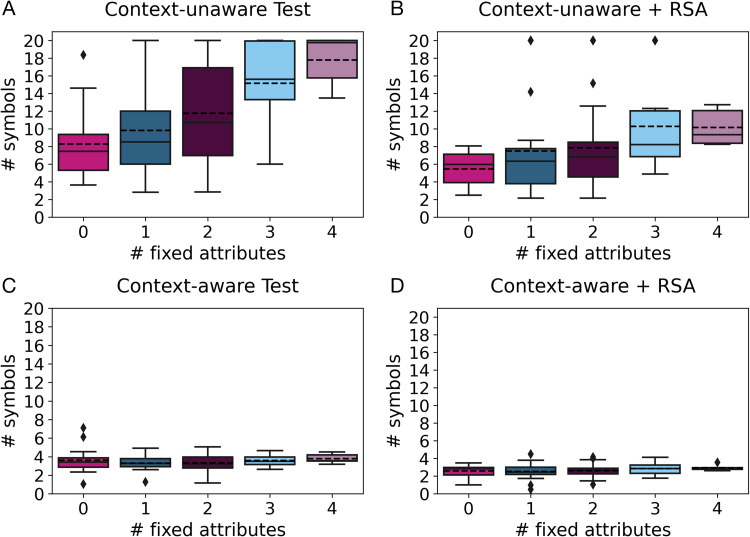
Distribution of message lengths for different levels of the conceptual hierarchy.

Next, we looked at the efficiency of the messages in relation to the amount of information that needed to be communicated, i.e., the number of fixed attributes in a concept (Fig 6A–6D). In the context-unaware condition (Fig 6A), the mean length of messages used to refer to generic concepts was 10.5 and the mean length of messages used to refer to specific concepts was 17.2. When adding RSA (Fig 6B), messages in the context-unaware condition became much shorter, i.e., they used messages of 5.5 symbols on average when referring to generic concepts and messages of 10 symbols on average when referring to specific concepts. In the context-aware condition (Fig 6C), message lengths were very short in general. In reference to generic concepts, context-aware agents used messages with 3.6 symbols on average and messages with 3.9 symbols on average in reference to specific concepts. Adding RSA reduced the mean lengths of messages used by context-aware trained agents by about 1 symbol in reference to both specific and generic concepts (Fig 6D). Overall, RSA improved efficiency and lead to shorter messages being chosen by the speaker agents. These observations were in line with the predictions of a Bayesian hierarchical model predicting message length by the factors context-based pragmatics (context-unaware vs. context-aware), utility-based pragmatics (without RSA, vs. with RSA) and conceptual hierarchy level (number of fixed attributes). We found a main effect for context-based pragmatics (M = 1.88, CrI=[1.28, 2.46], pd = 100%, ROPE=[-0.52, 0.52], 0% in ROPE), a likely existing main effect with undecided significance for utility-based pragmatics (M = 0.70, CrI=[0.09, 1.30], pd = 98.78%, ROPE=[-0.52, 0.52], 27.82% in ROPE), an interaction effect between context-based pragmatics and conceptual hierarchy (M = 0.87, CrI=[0.58, 1.18], pd = 100%, ROPE=[-0.52, 0.52], 0% in ROPE) and a possibly existing three-way interaction between context-based pragmatics, utility-based pragmatics and conceptual hierarchy with undecided significance (M = 0.28, CrI=[-0.02, 0.59], pd = 97.02%, ROPE=[-0.52, 0.52], 97.05% in ROPE).

Next, we looked at the lexical efficiency of the concept-message mappings. We calculated lexicon sizes and informativeness on the test interactions, i.e., the messages that have been produced when agents have been presented with concepts in the test dataset. The informativeness score is defined under Evaluation in the Methods section. Table 5 shows the number of concepts in the test datasets and the number of unique messages produced in the four different conditions. In a hierarchical Bayesian model predicting lexicon size by context-based and utility-based pragmatics and their interaction, we found no main effect for context-based pragmatics (M = -9.18, CrI=[-26.92, 8.37], pd = 84.67%, ROPE=[-26.42, 26.42], 99.68% in ROPE), i.e., no difference between context-unaware and context-aware training with regards to the lexicon sizes. However, we observed a main effect of utility-based pragmatics (M = 89.59, CrI=[71.74, 107.88], pd = 100%, ROPE=[-26.42, 26.42], 0% in ROPE) suggesting that RSA leads to smaller lexicon sizes. An interaction effect between context-based and utility-based pragmatics (M = -41.42, CrI=[-58.62, -24.36], pd = 100%, ROPE=[-26.42, 26.42], 2.00% in ROPE) that was probably significant, suggested that adding RSA reduced lexicon sizes mainly for context-aware trained agents but not for context-unaware trained agents.

**Table 5:** Lexicon sizes.

Dataset	# concepts	Context-unaware	+ RSA	Context-aware	+ RSA
(3,4)	250	99.6 ± 4.51	51.6 ± 4.34	153.8 ± 32.34	42.4 ± 5.55
(3,8)	1460	517.2 ± 10.13	379.8 ± 28.55	902.4 ± 116.91	155.4 ± 14.86
(3,16)	100	24.6 ± 9.4	56.4 ± 9.99	40.0 ± 17.39	39.0 ± 9.77
(4,4)	1250	736.6 ± 38.81	265.0 ± 63.53	821.6 ± 117.53	155.6 ± 25.21
(4,8)	100	42.0 ± 17.25	79.4 ± 3.05	66.4 ± 24.5	54.4 ± 6.02
(5,4)	100	43.8 ± 13.59	52.6 ± 21.7	82.6 ± 12.3	51.4 ± 4.1

Mean number of unique messages and standard deviations over five runs are calculated for each dataset.

**Fig 7:**
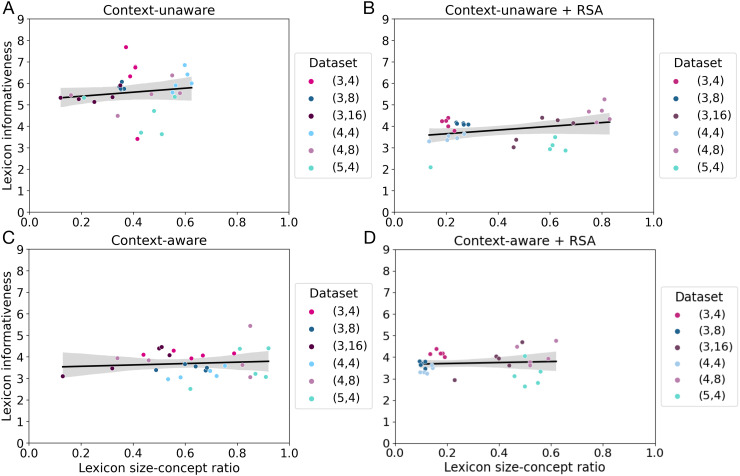
Tradeoff between lexicon size and informativeness. The lexicon size is normalized by the number of concepts in a dataset.

How did agents in the different conditions optimize the tradeoff between lexicon informativeness and size? In Fig 7A–7D, we plot the tradeoff between lexicon informativeness and size for each condition. We calculated size-concept ratios by dividing the number of unique messages sent by the number of unique concepts in the dataset. In the context-unaware condition (Fig 7A), the messages that the agents sent were very informative, i.e., they singled out specific concepts. No more than six messages were needed to refer to ten concepts on average. The lexicon of context-aware agents was less informative than that (Fig 7C): Lexicon informativeness was much lower and ranged from 3.21 to 4.11 for the different datasets. In a Bayesian hierarchical model predicting lexicon informativeness by context-based pragmatics (context-unaware vs. context-aware), utility-based pragmatics (without RSA vs. with RSA) and their interaction, we found a main effect for context-based pragmatics (M = 0.50, CrI=[0.39, 0.61], pd = 100%, ROPE=[-0.11, 0.11], 0% in ROPE), a main effect for utility-based pragmatics (M = 0.43, CrI=[0.32, 0.54], pd = 100%, ROPE=[-0.11, 0.11], 0% in ROPE) and an interaction effect (M = 0.44, CrI=[0.33, 0.55], pd = 100%, ROPE=[-0.11, 0.11], 0% in ROPE). These effects support the above observations. The mean lexicon size-concept ratios for each dataset were smaller in the context-unaware than in the context-aware condition. However, when adding RSA, this pattern was reversed (Fig 7B and 7D). In a Bayesian hierarchical model predicting lexicon size-concept ratio by context-based pragmatics (context-unaware vs. context-aware), utility-based pragmatics (without RSA vs. with RSA) and their interaction, we found a main effect for context-based pragmatics (M = -0.03, CrI=[-0.04, -0.01], pd = 99.90%, ROPE=[-0.02, 0.02], 33.63% in ROPE) with undecided significance, a main effect for utility-based pragmatics (M = 0.08, CrI=[0.06, 0.09], pd = 100%, ROPE=[-0.02, 0.02], 0% in ROPE) and an interaction effect (M = -0.09, CrI=[-0.10, -0.07], pd = 100%, ROPE=[-0.02, 0.02], 0% in ROPE). These effects suggested that adding RSA improved the lexicon size-concept ratio for context-aware but not for context-unaware agents.

**Fig 8:**
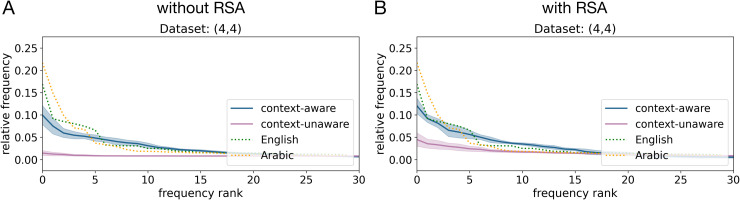
Zipf’s law like distribution of message frequency plotted for dataset D(4,4). D(4,4) is a medium-sized dataset where objects consist of four attributes which can each take four different values.

Lexicon-level efficiency of a language can also be quantified by how well the language follows a Zipf’s law like distribution of messages. The first part of Zipf’s law states that an efficient language contains few expressions which are used very frequently in a language [17]. In Fig 8A–8B, we present the frequency distribution of the messages that have been sent in the four different game scenarios, context-aware and context-unaware with and without RSA for one medium-sized dataset. Frequency distributions for all datasets can be found in S2 Fig and S3 Fig. Messages were ordered by their frequency rank on the x-axis and their relative frequency in the protocol is presented on the y-axis. We observed that in the context-unaware baseline, almost all messages were used with similar frequency. In the context-aware condition, some messages were used with very high frequency compared to the rest of the messages leading to the characteristic logarithmic relationship between relative frequency of messages in the protocol and their respective frequency rank. Adding RSA moved the respective distributions closer to a distribution resembling Zipf’s proposed relationship and natural languages, specifically the frequency distributions of English and Arabic corpora. The distribution of context-aware + RSA is closest to natural language. We conclude that the more we allowed for pragmatic mechanisms to be exploited in the simulations, the more the message distribution resembled a Zipf’s law like distribution of messages where there were few messages which were used very frequently and many messages which were used infrequently.

**Fig 9:**
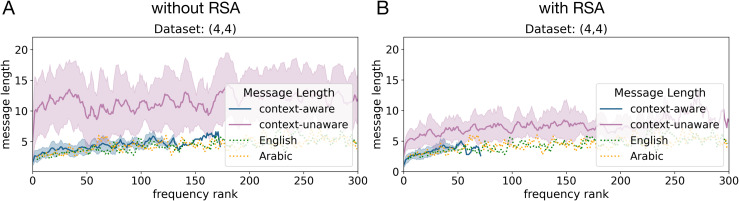
Zipf’s law like distribution of message length plotted for dataset D(4,4).

The second part of Zipf’s law states that languages maximize their efficiency by using shorter expressions more frequently than longer expressions [75]. In Fig 9A–9B, we present the distribution of messages as a relation between frequency rank on the x-axis and message length on the y-axis. We observed again that the more we allowed for pragmatic mechanisms to be exploited in the simulations, the more the message distributions resembled Zipf’s law and natural languages with shorter messages being used more frequently than longer messages.

Lastly, we present a qualitative analysis of messages that were used in the four different test conditions, context-unaware, context-unaware + RSA, context-aware and context-aware + RSA. Table 6 shows examples for the randomly picked specific test concept (2,0,3) in dataset D(3,4), which is the smallest dataset where objects have three attributes that can each take four different values. We use a tuple notation for concepts, e.g., (2,0,3), where only fixed attributes are specified and attributes which are not fixed by a concept but can take any value are represented as “_”, e.g., (2,_,0). For a more intuitive interpretation of the results, we give natural language examples. For example, (2,0,3) could mean small blue circle if the attributes were size, color and shape. These natural language examples are only possible interpretations of the concepts, the agents were trained on symbolic object vectors specifying only numerical attributes. We show examples for a coarse context, where the concept has been presented in a context where the distractor objects differed from the target concept in each attribute and a fine context, where the distractor objects in the context shared all but one attribute with the target concept. We list the messages that were sent and analyze their former use during training as well as which attributes of the concept the message probably communicated. In the context-unaware condition, the speakers are not aware of the context. This means that to be communicatively successful, they should communicate all attributes that are relevant to the target concept. Indeed, the message that was used to refer to (2,0,3) (SMALL BLUE CIRCLE) during testing (“1,1,1,1”) was used to refer to similar specific concepts during training, such as (2,1,3) (SMALL RED CIRCLE) and (2,3,3) (SMALL GREEN CIRCLE), or to refer to related more generic concepts, i.e., (2,_,3) (SMALL CIRCLE) and (2,0,_) (SMALL BLUE). We observed no difference between the coarse and the fine condition in the context-unaware baseline. When adding RSA to the context-unaware baseline, the messages tended to be shorter and a more diverse set of messages was used. This was likely due to different aspects of the target concept being communicated. We deduced this from the usage of the messages during training. By looking at the set of concepts that a message was used to refer to and their similarity, we reasoned about which attributes of the concept were likely communicated with this message. In the context-aware condition, we observed that the agents used different messages depending on the context condition (fine vs. coarse) and the specific distractor objects in the context. A likely strategy seemed to be that the agents compared the targets and distractors and communicated the one attribute that differed in the fine context condition or just one of the attributes that differed between targets and distractors in the coarse context condition. In the context-aware + RSA condition, agents used very similar messages to the messages that were used in the context-aware condition. The only difference was that they tended to use shorter messages where possible and, for example, reduced the messages “1,1,1,1” and “1,1,1” to “1,1”. Note that sending the message “1” would in this case be even more efficient, but not possible in this scenario because the RSA agents are only able to use messages that have been produced during training at least once.

**Table 6:** Qualitative examples.

Game scenario	Fixed indices	Fixed values	Context Condition	Distractors	Communicated attributes	Messages	Use during training
Context-unaware	(1,1,1)	(2,0,3)	0 (coarse context)	(1,3,0)	(2,0,3)	[1,1,1,1]	(2,1,3),(2,3,3)
				(0,1,0)			(2,_,3),(2,0,_)
				(0,1,1)			
			2 (fine context)	(0,0,3)	(2,0,3)	[1,1,1,1]	see above
				(2,3,3)			
				(2,1,3)			
Context-unaware + RSA	(1,1,1)	(2,0,3)	0	(1,3,0)	(2,0,_)	[1,2,1]	(2,0,_)
				(0,1,0)	(_,_,3)	[1,1]	(0,2,3),(0,_,3)
				(0,1,1)	(_,_,3)	[1,1]	(2,3,3),(2,_,3)
			2	(0,0,3)	(2,_,_)	[1,4,1,3]	(2,1,_)
				(2,3,3)	(_,0,_)	[2]	(1,0,3),(1,0,_)
				(2,1,3)	(_,0,_)	[2]	(_,0,_)
Context-aware	(1,1,1)	(2,0,3)	0	(1,3,0)	(2,_,_)	[4]	(2,_,_)
				(0,1,0)	(2,_,_)	[4]	
				(0,1,1)	(_,0,_)	[1]	None
			2	(0,0,3)	(2,_,_)	[4]	
				(2,3,3)	(_,0,_)	[1,1,1,1]	(_,0,_)
				(2,1,3)	(_,0,_)	[1,1,1]	(_,0,_)
Context-aware + RSA	(1,1,1)	(2,0,3)	0	(1,3,0)	(2,_,_)	[4]	(2,_,_)
				(0,1,0)	(2,_,_)	[4]	
				(0,1,1)	(2,_,_)	[4]	
			2	(0,0,3)	(2,_,_)	[4]	
				(2,3,3)	(_,0,_)	[1,1]	(_,0,_)
				(2,1,3)	(_,0,_)	[1,1]	

Qualitative examples for all four conditions and the dataset D(3,4) in response to the specific concept (2,0,3) (all attributes fixed).

## Discussion

Overall, the results of Experiment 2 suggest that in novel communicative situations, the agents that had been trained in the different context conditions in Experiment 1, used different approaches to communicate the novel concepts and did so with varying degrees of success. The strategies of context-aware agents and context-aware + RSA agents lead to almost optimal communicative success, whereas context-unaware and context-unaware + RSA agents exhibited lower accuracies on the test dataset. These differences in performance were likely due to the different strategies that were used: Context-unaware speakers which had been trained without access to a shared context, came up with a strategy where they tended to communicate all fixed attributes of a target concept as predicted. This was shown by the qualitative analysis of messages and has implications for the efficiency of the language used in this condition. Context-unaware agents used a smaller but very informative lexicon compared with the context-aware agents because they did not adapt their messages to the context conditions. Their messages tended to be longer, the more attributes were fixed in a target concept, suggesting that they communicated more attributes when the target concept was more specific. While such a trend is not the only conceivable outcome, it makes sense for the kinds of compositional concepts the agents learn to communicate in our setup. This is due to our manipulation of communicative needs: Our setup uses compositional concepts which need to be discriminated based on single attribute features from related concepts which often lies on the same conceptual hierarchy. A communication strategy which utilizes specific symbols to refer specific attribute features is thus useful because they can be combined and used flexibly in different messages to refer to different but related concepts. According to the communicative needs hypotheses, the messages that are lexicalized should reflect the discriminations that frequently need to be communicated by the agents during training.

The messages used in the context-unaware condition did not follow Zipf’s law, i.e., frequently used messages were not shorter nor used in more communicative situations. When adding utility-based pragmatic abilities in the form of RSA, context-unaware speakers sent messages that were more efficient and shorter. This effect was more pronounced when agents had been trained context-unaware than when they had been trained context-aware. This was likely due to the context-unaware condition being the one in which messages were very long in the first place. Even though the messages produced by context-aware speakers were already almost optimal with regards to their message length, adding RSA still improved the efficiency of the messages chosen by context-aware speakers that reasoned about their interlocutor’s likely interpretation with RSA. That RSA improved the efficiency of messages is unsurprising given that the utility function contains a cost term that penalizes longer messages. When both the informativeness of a lexicon and its size-concept ratio decrease, the lexicon can be said to be more efficient. However, when the informativeness decreases and the size-concept ratio increases, then the lexicon is not only less efficient but also less informative. This might explain why adding RSA in the context-unaware condition led to worse accuracies. Despite these performance issues, context-unaware speakers made more efficient use of their language when they reasoned about their interlocutor’s likely interpretation of their message and observed a cost on longer messages in the context-unaware + RSA simulations. This was also confirmed in the qualitative analysis where we saw that context-unaware agents chose different messages depending on the context. This adaptiveness to the context is due to RSA speakers reasoning about the listener’s likely interpretation of their message, an interpretation which happens in context. With RSA, the messages of context-unaware speakers resembled Zipf’s law like distributions a little more but still fell short of the efficiency of human languages where a logarithmic relationship is typically observed.

When communicating novel concepts, context-aware agents exploited a strategy that they cultivated during training and that allowed them to produce very efficient messages: They took the shared context into account and produced messages that would be ambiguous if taken out of context but could be unambiguously interpreted by the listener agent which observed the same context. This lead to very efficient messages in terms of message length, where only very few symbols were needed to communicate a target concept. The qualitative analysis shows that this was likely due to a strategy where the speakers strategically compared the target concept with the context and communicated only one attribute that differed between target concept and distractor objects. This communication strategy is very efficient as the agents only ever have to communicate one attribute. This strategy led to a set of messages that was less efficient than the set of messages that context-unaware trained speakers used in terms of the lexicon size and informativeness. This was likely due to the context-aware agents frequently using messages that contain redundancy. For example, the messages “1,1,1,1” and “1,1,1” from the qualitative analysis seem to have the same meaning, but would be counted as distinct messages in our calculation of the lexicon sizes. However, when adding RSA, the context-aware speaker agents were better able to exploit their efficient strategy and reduce redundancy in their messages which lead to smaller lexicon sizes. The lexicon informativeness was generally lower than in the context-unaware baseline. This makes sense because we have seen that the lexica contain more many-to-one mappings between messages and concepts in the context-aware condition which receive a lower informativeness score by definition. In the context-aware condition, adding RSA better optimized the tradeoff between lexicon informativeness and size-concept ratio than in the context-unaware condition. Context-aware speakers with RSA used messages which were as informative as the ones used without RSA, but they also used fewer distinct messages. Thus, it can be concluded that adding RSA had a more positive effect on lexicon efficiency in the context-aware than in the context-unaware condition. In terms of lexical efficiency, both context-aware and context-aware + RSA agents used a message distribution that closely resembles the Zipf’s law like distribution of human languages, where we observed two related phenomena: On the one hand, there was a small set of messages which was used very frequently while other messages were used only occasionally. On the other hand, messages which were used very frequently tended to be shorter than messages that were used less frequently. These results can be interpreted in light of both efficiency and pragmatic considerations: The more pragmatic mechanisms the agents can exploit, the more efficient use they can make of their emergent language.

## General discussion

We conducted two experiments to study the influence of the availability of pragmatic mechanisms, specifically context-based and utility-based pragmatics, on 1) an emerging language and on 2) the use of the emergent language in novel communicative situations. We showed that the availability of context-based pragmatics in the form of a shared context between interlocutors makes an emerging language more ambiguous and efficient. The prime mechanism for this efficiency is that the language’s ambiguity can be resolved in context. It emerged as a tradeoff between speaker and listener interactions where due to a more ambiguous language, message uncertainty is low for the speaker, while meaning uncertainty remains low for the listener. When these emerged languages are used in novel communicative situations, we found that the languages that emerged in the context-aware condition generalize better and are more efficient in terms of message length, while being less efficient in terms of lexicon size. The lexicon-level efficiency is, however, improved, when agents which have been trained context-aware are equipped with utility-based pragmatic reasoning during inference. In general, utility-based pragmatic reasoning improves the efficiency of the language and makes the message distributions look more like human natural languages.

In related work, Ekila et al. introduced a symbolic modeling approach including a novel concept representation as weighted feature channels. Despite the modeling approach and concept representation differing from our model, they arrive at similar results and conclusions about the general game dynamics such as that the agents can come up with robust, and coherent linguistic conventions which are adaptable to changes in communicative needs in the environment [52]. Another related line of research is the analytical approach by Brochhagen [76,77]. The main findings are that ambiguous signaling is adopted in informative contexts. Interestingly, contextual priors do not necessarily need to align between speakers and listeners. Rather, an ambiguous strategy is adopted when the speaker can anticipate the listener’s interpretation with utility-based reasoning. This tendency seems to be supported by an informative context in their work which is in line with our finding that languages that evolved under context-based pragmatics can be better exploited in utility-based pragmatics [77]. Our approach goes beyond such analytical approaches by providing evidence that — given pressures towards informativity and efficiency — ambiguity can emerge naturally from interactions between agents. This adds to the argument on the efficiency of ambiguity made in the literature and adds evolutionary plausibility to it [14,16,77].

In the following, we will first summarize the implications of our study for the emergent communication literature. Then, we will review in more general terms what can be gained for the field of pragmatics from using emergent communication models. After that, we will discuss the findings of experiments 1 and 2 for the emerging language with regards to the efficient communication hypothesis. After this language-level discussion, we will focus on the implications of our findings for category structure and categorization.

### Emergent communication as a framework to study pragmatics

This paper brings together two lines of research that do not traditionally interact (but see [13,37,78], for example). On the one hand, the emergent communication paradigm is used to investigate the emergence of novel communication systems between agents and general mechanisms of language evolution, e.g., [35,79,80]. On the other hand, the field of pragmatics is concerned with the role of context and utility-based pragmatic mechanisms in concrete communicative situations and repeated turns, e.g., [26,30,33,81]. In this paper, we brought these two lines of research together to ask what the role of pragmatics is in the emergence and evolution of a language. Our hypothesis was that pragmatics is a driving force on language emergence due to previous work showing the important role of context in language evolution. At the same time, pragmatics is also needed after the language emerged to achieve successful communication. Our setup allowed us to systematically investigate both context-based and utility-based pragmatic mechanisms on the two timescales of situational language use and language emergence.

Research on language evolution has long acknowledged the important role of pragmatics in shaping the communicative interactions, and, ultimately, the evolution of languages. For example, it has been shown that the context in which the individual interactions take place, shape an evolving language [8,39,49]. Previous work provided evidence that also emergent communication systems between artificial neural network agents are shaped by context [37]. Another important finding we build on is that the communication of hierarchically structured concepts shapes the emergent communication which is in line with the communicative needs hypothesis outlined in the introduction [37,38,41]. Our experiments add two novel dimensions to research on emergent communication: First, we do not only investigate the role of context-based pragmatic mechanisms, but also the role of utility-based pragmatic mechanisms traditionally considered in the field of pragmatics, in the emergence of novel communication systems. Second, we put more focus on investigating the emerged languages in novel communicative interactions to see more directly which features of the emergent language are especially useful for (future) communication.

Crucially, one of our main findings was that pragmatic mechanisms influence languages that emerge in non-human systems in a way that makes these languages more human-like. This suggests that the same pressures and pragmatic mechanisms might have shaped human language. Thus, we argue that we can not only learn something valuable about the driving forces on emergent languages between artificial agents as outlined in the discussions of Experiment 1 and 2, but that we can also learn something valuable about the role of different pragmatic mechanisms in the evolution and use of human languages. The following discussion reviews and discusses these learnings.

### The role of context in language evolution

In Experiment 1, we found that if speakers can take the context of their message into account, they are more likely to produce messages which are interpreted correctly by the listener, i.e., that are more likely to achieve communicative success. As a difference in performance between context-unaware and context-aware conditions was not found in an earlier comparison of context-aware and context-unaware trained agents [37], this is likely due to the shared context we introduced here which seems to help the agents to come up with a mapping between messages and concepts that also generalizes very well to the test dataset. This mapping is characterized by ambiguity, especially one-to-many mappings of messages to concepts, and high efficiency. Messages sent by context-aware agents tend to be shorter. This is likely due to the fact that it is not necessary for a context-aware speaker to communicate all fixed attributes in a concept. Rather, context-aware agents can communicate fewer attributes and let uncertainty about the message be disambiguated by the context. This leads to a more ambiguous language with more many-to-one and more one-to-many mappings between messages and concepts in the emerging protocol, suggesting that there is high signal and meaning uncertainty. Interestingly, this does not lead to a drop in performance. Together, these results suggest that context-aware agents develop a very efficient mapping between messages and concepts that makes use of the context for disambiguation.

Piantadosi et al. [16] have argued on the basis of Zipf [17] and Levinson [21] that inference is cheaper than reference and that ambiguity in language is the result of minimizing the communicative effort in a tradeoff between speakers and hearers. Following this argument, a language should evolve towards placing more burden on the listener (in the form of inference) than on the speaker (in the form of explicit and inefficient language). In our analyses, we used information-theoretic scores to quantify the tradeoff between speaker and hearer effort. Polysemy in the form of one-to-many mappings between messages and concepts places more burden on the listener while being easy for the speakers [16]. We observe more polysemy in the context-aware compared to the context-unaware condition which makes the efficiency tradeoff more optimal from the speaker’s perspective in the context-aware condition. Conversely, the languages that emerge in the context-unaware condition are more optimal for the listener, exhibiting more one-to-one mappings and low meaning uncertainty. We can conclude that the languages emerging in the context-unaware condition are more optimal for the listener, and that the languages emerging in the context-aware condition are more optimal for the speaker. This provides evidence for the claim made by Piantadosi et al. [16]: We observe that ambiguity emerges when the context can be utilized during the communication of target concepts. Given that in language emergence simulations the main training objective is to maximize the joint communicative success, i.e., the chance that the listener selects the correct target objects, it is unsurprising that in our context-unaware baseline, the emerging language is not optimal for the speaker. Adding a shared context, however, is enough to foster the utilization of this context to create a more ambiguous and efficient language as predicted by the efficient communication hypothesis. Following the argument presented above, this might be due to inference being cheaper than reference.

However, it is not clear whether inference is always cheap. Contrary to this assumption, pragmatic reasoning, such as the derivation of implicatures, reasoning about an interlocutor and general Theory of Mind, is typically believed to bear a cognitive cost and be effortful and slow [61,82]. Whereas ambiguity resolution in context may be considered relatively cheap, utility-based pragmatic inferences as modeled in Experiment 2 and recursive inferences about the interlocutor may still be effortful, slow and costly [63].

### The role of utility-based pragmatics in language use

In Experiment 2, we compared the situational performance of speaker agents trained either with access to the context or without access to the context, that either could or could not reason about the listeners’ likely interpretation of their message. We found that both context-based and utility-based pragmatics improve generalization performance. Adding RSA, i.e., utility-based pragmatics, improves the agents’ ability to generalize to novel concepts only when their language has emerged in a shared context. In line with our predictions, RSA-based reasoning leads to a better tradeoff between lexicon informativeness and lexicon size-concept ratio when agents have been trained context-aware. Overall, adding utility-based pragmatics modeled with RSA seems to be especially beneficial for agents trained in the context-aware condition. This might suggest that utility-based pragmatic reasoning hinges on similar mechanisms as context-based reasoning. Observing the effect of RSA-based reasoning in context is in line with experimental paradigms and theoretical models in the RSA literature [83]. While it is unsurprising that adding RSA improves the message-level efficiency due to the cost term (in the RSA implementation), we show that adding RSA also improves lexicon-level efficiency an emergent feature that was not directly encoded in the utility function. One reason for the improved lexicon-level efficiency might be that when messages are shortened, their meanings are summarized. For example, we have seen in the qualitative analysis that context-aware agents have used messages of varying lengths that contained only one symbol. Context-aware + RSA agents were able to shorten the messages and reduce symbol redundancy. The meanings of the messages of varying lengths were thus collapsed into the shortest possible message.

In comparison to human natural languages such as English and Arabic, we find that the agents trained in the context-aware condition make efficient use of their languages during inference. Specifically, we show that the more pragmatic mechanisms become available to the agents, i.e., context-based and utility-based pragmatics, the more the distribution of messages resembles Zipf’s law in terms of frequency and message lengths. The hypothesis that Zipf’s Law of Abbreviation arises from the Principle of Least Effort, i.e., due to pressures of accuracy and efficiency, has been tested experimentally in an artificial language learning study with human participants [7]. Here, we provide further evidence for this claim and show that even in our agent-based model, a Zipf’s law-like distribution emerges from the same pressures, i.e., the training objective to achieve communicative success and the efficiency pressure of keeping messages short.

In our model, we implement RSA-based reasoning for the speaker at inference, i.e., during testing on novel concepts [57]. This allows us to investigate the use of an already emerged language in production. We focus on the production side due to two main reasons, one practical and one theoretical. The practical reason is that inferring over the space of messages uttered during training is tractable for our simulations, whereas a listener model would need to infer over the space of possible world states, which would heavily increase the computation time. In our simulations, we approximate the space of all possible messages by choosing training utterances only, i.e., by letting the RSA agents reason about utterances that have been produced previously. The theoretical motivation for investigating an RSA speaker is that much work in experimental pragmatics has focused on comprehension due to the difficulties in setting up controlled production experiments. We can thus add to the body of literature focusing on production. Our model implements RSA-based reasoning in the speaker model by simulating an internal listener that the speaker reasons about. This approach is not too far from what speakers might actually be doing in conversation: Speakers might, when choosing a referring expression, think about how they would interpret this referring expression as a listener in the same context.

### Communicative efficiency as a tradeoff between speakers and listeners

In our experiments, we investigated the emergence and use of languages in an agent-based setup where agents either had no pragmatics, only context-based pragmatics, only utility-based pragmatics, or both context- and utility-based pragmatics to their availability. In our model, the tradeoff between speaker and listener needs as hypothesized by the efficient communication hypothesis, is implemented in interactive communication. Our research goes beyond previous work investigating the efficiency tradeoff between speakers and listeners in a small-scale probabilistic model [11]. Our work extends on this research by showing that the idea of an efficiency tradeoff can scale up to more complex worlds and larger vocabularies than the limited utterance and meaning space considered in [11]. Another benefit is that we can show that the influence of the availability of a shared context is an emergent feature: The availability of a shared context alone drives its use in communication and influences the emerging language towards more ambiguity, efficiency and better communicative success. The results of our experiments suggest that the efficiency tradeoff between speakers and listeners is dependent on the availability of context. In the context-unaware condition, languages are more optimized from the listener perspective, providing messages with unique meanings. In the context-aware condition, languages are more optimized for the speakers with polysemous messages that are efficient and require listeners to disambiguate meanings in context.

This is in line with experimental evidence that suggests that there is an imbalance between the use of pragmatic reasoning between speakers and listeners. For example, Baumann et al. [59] have investigated free productions of referring expressions in reference games. They found that in reference situations in which a shorter utterance would require pragmatic reasoning, speakers use utterances that require pragmatic reasoning in only about half of the trials. In the other half, speakers produce messages that describe more target features than necessary to avoid the need for pragmatic reasoning. This is despite the fact that listeners are able to use pragmatic reasoning to derive the correct interpretation. Relatedly, Ferreira [29] reports evidence that speakers rarely avoid ambiguity and leave much of the required labor for the listeners who need to disambiguate the utterance’s meaning. We find that the same pattern emerges between speaker and listener agents in our simulations in the context-aware condition. Together, this evidence seems to suggest that it is not detrimental to communicative success if speakers use the more ambiguous and efficient utterances and place more burden on the listener. Instead, this seems to be an emergent feature of languages that evolve in a shared context.

Coming back to the question of whether utility-based pragmatic reasoning about the interlocutor’s likely interpretation is slow and effortful as opposed to context-based pragmatics which is regarded as fast and effortless [63], the results of our simulations can make a contribution to this debate. While we cannot draw inferences about processing costs on the listener side from our simulations, we can examine the communicative success and production efficiency. We demonstrate that utility-based pragmatic reasoning has a more positive effect on communicative success and generalization performance if agents had been trained within a shared context than if they had been trained without a shared context. In other words, languages that emerged in an informative context are better suited to support utility-based pragmatic reasoning on the speaker side. This suggests that it is inefficient to try and decouple context and pragmatic reasoning. Thus, we would expect that if the context is informative, the effort of engaging in utility-based pragmatic reasoning on the speaker side should be low (or can be avoided altogether as suggested by recent experimental findings [59]).

### Implications for conceptual structure

The emergence of ambiguity, specifically one-to-many and many-to-one mappings between messages and concepts, in the context-aware condition is tightly linked to the way speakers refer to concepts at different levels of abstraction, i.e., to more specific or more generic concepts. This suggests that in our simulations, category structure and language are coupled. Specifically, we observe differences in referring to more specific vs. more generic concepts: There are only few messages that are available for referring to a generic concept but multiple messages that are available for referring to a specific concept. Similarly, the messages used to refer to generic concepts typically uniquely identify the target concept, whereas the messages used to refer to specific concepts could also be used in reference to more than one concept. These results suggest that one reason for why languages have many-to-one and one-to-many mappings is closely tied to conceptual structure. Specifically, communication about the hierarchically organized concepts in our simulations lead to the emergence of a language that does not only encode hierarchical concepts, but the one-to-many mappings of which also lets the agents use the same name for several concepts in the same conceptual hierarchy (such as “animal” in natural language) and the many-to-one mappings of which lets the agents use several names from the same conceptual hierarchy (such as “animal”, “dog”, “dalmatian”) for the same concept. These findings connect to the literature that investigates the influence of efficiency on the evolution of category systems such as color and kinship in language [13,23,84].

The number of different messages that can be used to describe the same concept increases exponentially in the context-aware, but not in the context-unaware condition. This makes languages that emerged in the context-aware setting likely more similar to natural languages, where concepts are thought to be organized in a hierarchical structure [85]. We conclude that the availability of context not only shapes a language emerging from referential communication, but it also shapes how this language encodes concepts, i.e., the category structure and the process of categorization. This is in line with the idea that context helps in category learning [85].

## Conclusion

In conclusion, we have shown that context-based and utility-based pragmatics play a role in referential communication and categorization by shaping an emerging language. While pragmatics is not necessary for the emergence of a meaningful mapping between messages and hierarchical concepts, we have provided evidence that the availability of a shared context between speakers and listeners shapes an emerging language to the effect that it becomes more efficient and that it generalizes more effectively. When context is available, the efficiency tradeoff between production and comprehension, i.e., between simplicity and informativeness, puts more burden on the listener side. This means that the communication system can become simpler by being less informative because listeners can reason about the context and resolve the meaning of polysemous messages in context. In turn, this does not happen when speakers are trained without access to a (shared) context. Hence, our results provide evidence for the role of pragmatics in shaping category structure and communication about categories. We can consider the relationship between environment, communicative need and the category system to be modified by the availability of context. As predicted by Rosch et al. [19,20], the category system of a language can afford to be simpler when the meanings of categories are interpreted in context.

In addition to the influence of the availability of context-based pragmatics on the emergence of a language, we investigated the role of utility-based pragmatics in novel communicative situations. We found that speakers choose more efficient messages when they reason about the utility of the message in terms of the listener’s likely interpretation via RSA-based reasoning. In summary, we have shown how context-based pragmatics shapes a language’s category system when concepts at different levels of abstraction need to be communicated in interaction. Utility-based pragmatics has been shown to be especially effective when used for an emerging language that includes ambiguity in the context-aware condition, and its main function in our model is to make the communication about concepts at different levels of abstraction more efficient from the speaker perspective. Our findings suggest that efficient category systems that are reflected in an efficient linguistic system can emerge in agent-based models of communication in interaction. This has important implications for both emergent communication between artificial agents and for the question how human category and language systems might have evolved and are continuously shaped by the environment and a communicative need: Pragmatics, in the form of context and utility-based reasoning, plays a crucial role in shaping communicative interactions and, ultimately, an evolving communication system.

## Methods

### Formalization

We use a multi-agent model of emergent communication. In emergent communication models, a speaker and a listener agent need to communicate about a target in a given context. In one round of the game, speaker and listener observe an input each, and then the speaker sends a message to the listener that describes the target. The listener interprets the message and selects the object they believe is the target, i.e., the one they assign the highest probability. During several iterations of training, the agents converge on a language-like system, i.e., a consistent mapping between targets and messages [35,36]. To prevent agents from communicating about lower-level features that are not relevant from a human perspective, we use a concept-level reference game for training [37,41]. This means that the agents do not communicate about a single object, but rather about a concept. We achieve this by creating concepts that consist of multiple target objects [37,41], see Fig 1.

The model is formalized as a communication game $G=(T^{S},D^{S},T^{L},D^{L})$ between a speaker agent *S* and a listener agent *L*. Both agents receive their own set of input objects, comprised of game size *g* target objects $T=\{t_{1},...,t_{g}\}$ and *g* distractor objects $D=\{d_{1},...,d_{g}\}$. The speaker agent receives their own sets of targets *T*^*S*^ and distractors *D*^*S*^ ordered such that targets come first. If a*g*ents are trained context-unaware, the speaker agent does not process the distractor objects, but only the target objects. The receiver agent receives their own sets of targets *T*^*L*^ and distractors *D*^*L*^, but they are shuffled and the listener does not know which objects are targets and which are distractors. This means they receive an input $X^{L}=\{x_{1}^{L},...,x_{i}^{L}\}$, where i=2·g. The individual objects that comprise the target concept and the context are not necessarily shared between listeners and speakers, but they belong to the same target concept and context condition. For example, if the target concept is BLUE, then what *T*^*S*^ and *T*^*L*^ have in common is that all objects in these sets are blue. However, it does not matter, whether the target objects are blue circles, blue squares, blue triangles etc. These are randomly sampled from the set of blue objects. Similarly, in a context condition where one concept-defining attribute is allowed to be shared between *T*^*S*^ and *D*^*S*^ and between *T*^*L*^ and *D*^*L*^, then the specific objects in the sets *D*^*S*^ and *D*^*L*^, may differ. However, both distractors presented to the speaker and distractors presented to the listener differ in the same concept-defining attributes from the target concept. For example, blue circles are presented in a context comprised of circles of different colors (see Fig 2B). The listener’s task is to predict a label $y_{i}^{L}\in \{0,1\}$ (0: distractor, 1: target) for each object $x_{i}^{L}$ based on a message *m* that it receives from the speaker. The speaker generates a message *m* by choosing up to *M* symbols from a vocabulary *V*. The vocabulary is comprised of primitive discrete symbols, ranging from 0 to *V*, i.e., “0,” “1,” “2,” etc. [36]. The symbol 0 is defined as the end-of-sequence (EOS) symbol that can be used to terminate a message before *M*, the maximum message length, is reached [37,38,41]. All other symbols can be sent without such implications. They do not have a meaning in the beginning of training, but their meaning is negotiated and converged upon through training, i.e., through several iterations of playing the concept-level reference game. We train the speaker and listener agents with a joint binary cross entropy (BCE) loss maximizing the probability that the listener agent correctly identifies the target objects in their input:


$$\begin{array}{c} {ℒ}_{BCE}(S,L,G)=-\sum_{i}\log p^{L}(y_{i}^{L}|x_{i}^{L},\hat{m}), \end{array}$$ (2)

where $\hat{m}\sim p^{S}(m|T^{S},D^{S})$ and $p^{L}(y_{i}^{L}|x_{i}^{L},\hat{m})=\text{ReLu}({\text{GRU}}^{L}(\hat{m})\cdot \text{embed}(x_{i}^{L}))$.

We implement the game using the EGG framework [86] developed for implementing emergent communication games. Both our speaker and listener agents are implemented as neural networks with feed-forward (dense) layers for embedding the input objects and Gated-Recurrent-Unit (GRU) [87] cells to encode (speaker) or decode (listener) the messages. GRU cells are a specific type of Recurrent Neural Networks (RNNs) which have been proven useful for dealing with sequence data such as language. The GRUs consist of a single hidden layer and a gating mechanism that weighs how much previous information is considered when processing the current input. They are thus well-suited for detecting dependencies and patterns in language-like input.

### Modeling a pragmatic speaker with the Rational Speech Acts (RSA) framework

We model an RSA speaker following the Rational Speech Acts (RSA) framework [33,34,88]. The RSA model provides a framework to model different listener and speaker types that vary by the information they take into account to choose or interpret a message. The base level speaker *S*_0_ chooses a message that describes the targets. The base level listener *L*_0_ interprets the message literally, i.e., it predicts which are the targets based on the learned meaning of a given message. The RSA speaker *S*_RSA_ we use in this model is a level-1 speaker *S*_1_, also called Gricean speaker [34], which maximizes relevant information as to allow the hearer to choose the correct target. This means that based on the assumption that a literal listener *L*_0_ chooses all targets of which the message is true with equal probability, the RSA speaker chooses a message that maximizes the probability of the listener to select the targets. For example, if the speaker needs to communicate a BLUE CIRCLE in a fine context consisting of CIRCLES of different colors than blue, then *S*_0_ would choose the messages “blue” or “circle” with equal probability. *L*_0_ would then choose targets of which the message is true, leading to communication success if the message was “blue” and to communication failure if the message was “circle”. An *S*_1_ speaker that we call *S*_RSA_ in our model, would choose the message “blue” with higher probability than “circle” because they reason about the listener’s likely interpretation of the message. We implement *S*_RSA_ as a speaker that maximizes a utility function following standard RSA models [33,34,88]:


$$\begin{array}{c} S_{\text{RSA}}(T^{S},D^{S})=\text{arg}\max_{m\in ℳ}U(m|T^{S},D^{S}), \end{array}$$ (3)

where *U* is the utility function, typically defined as $U(m|w)=\log p^{L_{0}}(w|m)$ [88]. This means that speakers are rewarded if they choose a message that maximizes the log-likelihood of listeners interpreting the world *w* correctly given message *m*. Often this basic utility function is refined, for example by adding a cost term that penalizes long messages [58]. In our implementation, we follow previous work [57] and define the utility function as:


$$\begin{array}{c} U(m|T^{S},D^{S})=\log p^{L}(y_{i}^{L}|x_{i}^{L},m)-C(m), \end{array}$$ (4)

where *C*(*m*) is a cost applied to the message length and $p^{L}(y_{i}^{L}|x_{i}^{L},m)$ is the log-likelihood of the listener to select the correct targets, i.e., to predict the correct labels *y*_*i*_ based on a message and input objects $x_{i}=(T^{S},D^{S})$. The speaker evaluates the utility based on an internal listener model which is the trained agent from the first experiment *L*_0_. We define $\log p^{L}(y_{i}^{L}|x_{i}^{L},m)$ as the logits output from the internal listener model averaged for targets and distractors. To account for the fact that in typical RSA models, the messages that maximize the probability of choosing the correct targets at the same time minimize the probability of selecting the distractors, we calculate the overall utility as $\frac{\sum y_{i}=1}{\left|y_{i}=1\right|}-\frac{\sum y_{i}=0}{\left|y_{i}=0\right|}$. Importantly, the RSA speaker *S*_RSA_ only has access to their targets *T*^*S*^ and distractors *D*^*S*^ and thus is only able to infer how the listener would interpret a message referring to the target concept the speaker observes in a given context. This preserves some naturalness of the communicative scenario by preventing speakers from being omniscious. Finally, the accuracy is calculated on how the listener actually interprets a message in their own context *x*_*i*_ consisting of *T*^*L*^ and *D*^*L*^. The cost function C(m)=λ|m| penalizes messages based on their length, i.e., the number of symbols in a message. We use a cost factor of 1 for our simulations.

### Dataset

**Fig 10:**
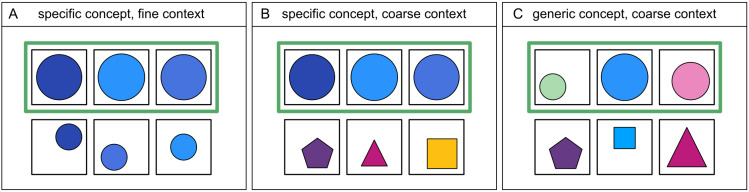
Dataset examples. The three objects in the top row are the target objects that form a target concept together. The three objects in the bottom row are the distractor objects that form the context. A: An example for a specific concept LARGE BLUE CIRCLE in a fine context (two attributes shared). B: An example for a specific concept LARGE BLUE CIRCLE in a coarse context (no attribute shared). C: An example for a generic concept CIRCLE in a coarse context.

We construct six symbolic datasets that contain concepts with varying levels of specificity (ranging from specific to generic) depending on how many attributes are shared within a concept, i.e., between the different target objects. Objects are symbolic vectors comprised of *n* attributes where each attribute can take *k* values. For example, (1,2,1) is an object from the dataset D(3,4). We use the notation D(*n*,*k*) to denote datasets of different sizes, ranging from three to five attributes which can each take between four and 16 values. Target objects that belong to a specific concept share all attributes. Target objects that belong to a generic concept share only one attribute. This means that the more specific a concept is, the more information needs to be communicated when describing the concept. Each concept is presented in a context. Depending on how many attributes are shared between the target objects and the distractor objects, we compare contexts ranging from fine contexts, in which all but one attributes are shared, to coarse contexts, in which one attribute is shared between targets and distractors. The context granularity also relates to how much information needs to be communicated to describe a given concept. As a general rule of thumb, the finer the context, the more information needs to be communicated. In Fig 10A-10C, we present examples for concept and context combinations in the datasets. We use shapes with different colors and sizes only for visualization purposes. The agents are trained on symbolic vectors as described above. 60% of the concepts are assigned to the train split and 20% of the concepts are assigned to the validation split of the dataset. Each concept is presented in exactly one context, where the number of shared attributes is randomly sampled and shared between speaker and listener inputs. The data split used for testing (20% of the data) contains novel concepts in randomly sampled contexts.

### Hyperparameters and training

We use the following hyperparameters: The agents have internal GRUs with a hidden size of 128 to encode and decode messages. We trained with a batch size of 16 and a learning rate of 0.001. The datasets were created with game size 10, i.e., 10 targets and 10 distractors per game round, and scaling factor 10, i.e., each concept is represented in the dataset 10 times. For training, we make use of the Gumbel-Softmax function that makes it possible to use backpropagation [89], a loss pressure that penalizes the length of messages and an early stopping criterion to make training more efficient. Details can be found in S1 Appendix.

### Evaluation

We evaluate the models and emerging languages according to the following criteria: First, we report accuracies on the train, validation and test datasets. Accuracies on the train and validation datasets give us a measure of communicative success, i.e., how well do the listener agents perform, or, in other words, how often do they select the correct target objects. Accuracies on the test datasets additionally can be used as a measure for generalization abilities. Here, we can see whether the emerging language generalizes well to unseen concepts.

Second, to investigate whether certain game scenarios lead to the emergence of more efficient languages, we need a measure that captures the efficiency of an emerging language. In the literature, efficiency has been related to small vocabulary sizes and to short messages lengths while communicating the same amount of information [16]. While the vocabulary size is in principle fixed in our simulations, we can measure the message lengths of an emergent language directly after training. We also calculate the number of unique messages used to refer to concepts as an approximation to the agents’ lexicon size [36]. We relate the size of the lexicon to the lexicon’s informativeness. The informativeness *I* of a lexicon *L* is defined as the average over the message informativeness *I*_*m*_ over *N* interactions following [13]:


$$\begin{array}{c} I_{L}=\frac{1}{N}\sum_{i=1}^{N}I_{m}^{i} \end{array}$$

with $I_{m}=\frac{1}{S_{m}}$, where


$$\begin{array}{c} S_{m}=\frac{1}{N}\sum_{i}\sum_{j\neq i}d(C_{i},C_{j}) \end{array}$$

is the spread of features that is calculated based on the average distance between concepts *C* that have been referred to by *m*. This means that messages receive a lower informativeness score if they are used to refer to concepts with higher distance and that messages receive a higher informativeness score if they are used to refer to concepts with lower distance, i.e., higher similarity. This captures the intuitive idea that words in a language which refer to a highly specific concept such as DALMATIAN are more informative than words which refer to a highly generic concept such as ANIMAL. A natural language lexical system contains both highly specific and highly generic references and optimizes the tradeoff between size and specificity.

Third, relating back to the proposed efficiency tradeoff between production and comprehension, we expect that an efficient language should contain ambiguity, i.e., one-to-many and many-to-one mappigns between messages and concepts. To measure whether an emergent communication protocol contains such ambiguous mappings, we calculate information-theoretic scores on the set of *K* messages $M=\{m_{1},...,m_{K}\}$ uttered and the set of *L* target concepts $C=\{c_{1},...,c_{L}\}$ the agents communicated about during the simulation. We calculate these scores on the final interactions between speakers and listeners in the last training epoch.

**Fig 11:**
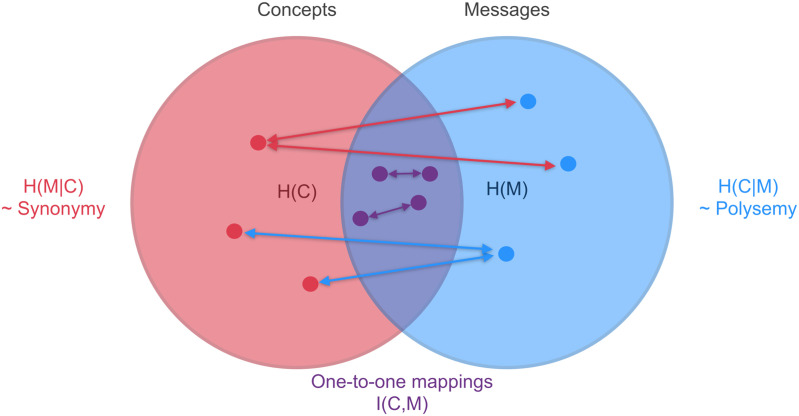
Information-theoretic scores and ambiguity in language. The set of concepts *C* is the red circle on the left (red and purple), the set of messages *M* is the full blue circle on the right (blue and purple). The mutual information *I*(*C*,*M*) (only purple) captures one-to-one mappings between messages and concepts. The conditional entropy H(M|C) (only red) captures many-to-one mappings between messages and concepts. The conditional entropy H(C|M) (only blue) captures one-to-many mappings between messages and concepts.

Fig 11 visualizes the sets of concepts and messages (displayed as circles) with instances of the concepts and messages displayed as dots. The relevant information-theoretic scores are calculated on these two sets.

A language that is maximally efficient only for the listener but not for the speaker should contain only one-to-one mappings between concepts and messages. The amount of one-to-one mappings in a language can be calculated with the normalized mutual information score between concepts and messages:


$$\begin{array}{c} NMI(C,M)=\frac{H(M)-H(M|C)}{0.5\cdot (H(C)+H(M))}. \end{array}$$ (5)

If this score is 1, then the efficiency tradeoff is maximal for the listener and minimal for the speaker. If this score is 0, on the other hand, we cannot conclude that efficiency for the speaker is high; as it might be that a completely random mapping between concepts and messages have been learned that would also be highly inefficient for the speaker. Thus, to address efficiency for the speaker, we consider how much ambiguity, i.e., how many one-to-many and many-to-one mappings, an emergent protocol includes. To do this, we calculate scores based on conditional entropies. The conditional entropy of messages given concepts,


$$\begin{array}{c} H(M|C)=-\sum_{c\in C,m\in M}p(c,m)\log\frac{p(c,m)}{p(c)}, \end{array}$$ (6)

measures how much uncertainty remains about the messages after knowing the concepts, i.e., the signal uncertainty. High signal uncertainty means that there is a many-to-one mapping of signals-to-meanings [50]. In other words, when knowing the concept, it is highly uncertain which message has been used to refer to it. This measure is used to capture the synonymy of messages [38,50]. In our case, synonymy can also reflect alternative descriptions for the same concept, such as when the same DOG can be referred to with the labels *dog* or *dalmatian*. The consistency score uses the conditional entropy H(M|C) to measure how much uncertainty remains about the messages after knowing the concepts. It is calculated as follows:


$$\begin{array}{c} \text{consistency}(C,M)=1-\frac{H(M|C)}{H(M)}. \end{array}$$ (7)

If the consistency score is maximal, i.e., 1.0, this means that the mapping between messages and concepts is highly consistent, or, in other words, that there are no many-to-one mappings between messages and concepts.

Conversely, the conditional entropy of concepts given messages,


$$\begin{array}{c} H(C|M)=-\sum_{c\in C,m\in M}p(m,c)\log\frac{p(m,c)}{p(m)}, \end{array}$$ (8)

measures how much uncertainty remains about the concepts after knowing the messages, i.e., the meaning uncertainty. High meaning uncertainty means that there is a one-to-many mapping of signals-to-meanings [50]. In other words, when knowing the message, it is highly uncertain which concept it refers to. This measure captures the polysemy of messages [38,50], or, in our case, if the same description of a concept, such as the superordinate label *animal*, can be used to refer to multiple concepts at other levels of the same conceptual hierarchy. The effectiveness score uses the conditional entropy of concepts given messages H(C|M) to measure how much uncertainty remains about the concepts after knowing the messages. It is calculated as follows:


$$\begin{array}{c} \text{effectiveness}(C,M)=1-\frac{H(C|M)}{H(C)}. \end{array}$$ (9)

If the effectiveness score is maximal, i.e., 1.0, this means that the language is highly effective, or, in other words, that there is no uncertainty about the concepts after knowing the messages, i.e., no one-to-many mappings between messages and concepts in the lexicon.

Fourth, as another measure of efficiency and to assess whether the emerging languages share efficiency related properties with human languages, we analyze the frequency distributions of messages. We investigate the following two relationships proposed by Zipf’s law [17,75] which seem to be a common principle of natural languages on the lexicon level [90–92]. First, when ordering messages according to their frequency, i.e., their rank in a lexicon, it has been shown that the relative frequency of a word in a corpus decays exponentially with each rank. This means that few messages are used very frequently [92]. Second, word lengths are determined by their frequency, where the most frequent words tend to be the shortest in a language [91]. We test these two properties on the agent’s protocols of messages used during inference on novel test concepts and compare the resulting distributions in the different conditions and to natural language data from English and Arabic. We use the Leipzig Corpora Collection [93] and specifically the News copora with 10K words for English (2024) and Arabic (2022) including words and their ‌‌frequencies in the corpus. Frequency ranks are determined by sorting the words/messages by their frequency of occurence $F=\{f_{1},...f_{m}\}$. The relative frequency *F*_*rel*_ of a word is calculated as


$$\begin{array}{c} F_{rel}=\frac{f_{i}}{\sum_{j}^{m}f_{j}}, \end{array}$$

where *m* is the number of words/messages and *i* is the *i*th word. We choose *m* = 30 for both artificial simulations and natural languages, i.e., we calculate the frequency distributions for the 30 most frequent words/messages. We calculate message lengths with a Python script and the PyArabic library [94].

## Supporting information

S1 Appendix Training specifics and hyperparameters. This text provides details on the training procedure using Gumbel-Softmax relaxation, a length cost pressure and early stopping.
(PDF)

S2 Appendix Statistical models. Three tables specify the model syntax and priors used for the statistical models fitted for Experiment 1 and 2.
(PDF)

S1 Fig Training trajectories. In S1 Fig, we show the training trajectories of five runs trained on the training and validation datasets of D(4,4) when training context-unaware (S1A Fig) and context-aware (S1B Fig) agents.
(TIF)

S1 Table Learning speed/ease of learning. S1 Table shows the mean number of epochs that were needed to train the agents for each dataset and condition.
(TEX)

S2 Fig Frequency rank distributions for all datasets. In S2 Fig, we present the frequency rank distributions for each dataset and for both context-unaware and context-aware trained agents without RSA (S2 FigA) and with RSA (S2 FigB).
(TIF)

S3 Fig Message length frequency rank distributions for all datasets. S3 Fig shows the message length frequency rank distributions for each dataset and for both context-unaware and context-aware trained agents without RSA (S3 FigA) and with RSA (S3 FigB). Curves are smoothed using a sliding average of five for better visibility. Arabic words have been stripped of diachritics with the library PyArabic [94] before calculating their length.
(TIF)

S3 Appendix Amount of ambiguous trials in the test dataset. This text identifies ambiguous trials in the test dataset and provides an RSA rationale.
(PDF)
