# Supplementary material for: The role of pragmatic mechanisms in referential communication and categorization: An emergent communication model
Source: PLoS Comput Biol. 2026 May 26;22(5):e1014326. doi: 10.1371/journal.pcbi.1014326 (PMC13252840; doi:10.1371/journal.pcbi.1014326)
Supplement: S1 Appendix — This text provides details on the training procedure using Gumbel-Softmax relaxation, a length cost pressure and early stopping. (PDF) [file pcbi.1014326.s001.pdf]

S1 Appendix

Training specifics and hyperparameters

A Gumbel-Softmax relaxation

We train with the Gumbel-Softmax relaxation that makes it possible to use backpropagation [1]. We use a temperature of 2.0 and a temperature update of 0.99.

B Length cost pressure

We train the agents with a loss pressure that penalizes the length of messages by multiplying the symbol's position in a message with a cost factor c_f. We choose the cost factor $c_f = 0.001$. We set the maximum message length M to 20 symbols and the vocabulary size V to the number of values in a dataset plus one for the end-of-sequence (EOS) symbol. There are two main reasons for applying such a length pressure during training. The first reason is that the length cost pressure works like an efficiency pressure on the emerging language. If we expect differences between the context-unaware and the context-aware scenario, then these should be due to efficiency reasons. While human communication is typically costly, with neural network agents sending bits of information is cheap and we thus need to include a pressure that incentivizes the agents to use efficient language. The second reason is that for the RSA simulations to work, we want the training to generate an emerging language that contains messages of different lengths. These can be better exploited by the RSA senders. If messages generated during training were all of the same length, then the cost term in the RSA utility function would have no effect.

C Early stopping

We use early stopping for training. We stop training when a validation accuracy of 0.90 has been reached at least once during training and the validation loss has not changed more than 0.001 during 10 epochs. In case these criteria are not met during 300 epochs of training, we stop nevertheless.

References

1. Jang E, Gu S, Poole B. Categorical Reparameterization with Gumbel-Softmax. In: International Conference on Learning Representations (ICML); 2017.Available from: `https://openreview.net/forum?id=rkE3y85ee`.
