# Supplementary material for: The role of pragmatic mechanisms in referential communication and categorization: An emergent communication model
Source: PLoS Comput Biol. 2026 May 26;22(5):e1014326. doi: 10.1371/journal.pcbi.1014326 (PMC13252840; doi:10.1371/journal.pcbi.1014326)
Supplement: S2 Appendix — Three tables specify the model syntax and priors used for the statistical models fitted for Experiment 1 and 2. (PDF) [file pcbi.1014326.s002.pdf]

S2 Appendix

Statistical models

In Tables A and B, we specify the model syntax and priors used for the statistical models fitted for Experiment 1 and 2, respectively. Table C presents the posterior summaries for the Bayesian hierarchical models predicting NMI, effectiveness and consistency for Experiment 1.

Table A. Statistical models fitted with brm for Experiment 1.

Model	Syntax	Priors
1) Accuracy	validation accuracy ~ context + (1|dataset)	`Intercept`:uniform(0, 1), `sigma`: uniform(0, 0.1)
2) Message length	message length ~ context * fixed attributes + (1|dataset)	`Intercept`:uniform(0, 20), `sigma`: uniform(0, 5)
3) Entropy	NMI ~ context + (1|dataset)<br>effectiveness ~ context + (1|dataset)<br>consistency ~ context + (1|dataset)	`Intercept`:uniform(0, 1), `sigma`: uniform(0, 0.1)
4) Entropy * concept hierarchy	NMI ~ context * fixed attributes + (1|dataset)<br>effectiveness ~ context * fixed attributes + (1|dataset)<br>consistency ~ context * fixed attributes + (1|dataset)	`Intercept`:uniform(0, 1), `sigma`: uniform(0, 0.1)

Table B. Statistical models fitted with brm for Experiment 2.

Model	Syntax	Priors
1) Accuracy	test accuracy ∼ context ∗ RSA + (1|dataset)	`Intercept`:uniform$(0, 1)$, `sigma`: uniform$(0, 0.1)$
2) Message length	message length ∼ context ∗ RSA ∗ fixed attributes + (1|dataset)	`Intercept`:uniform$(0, 20)$, `sigma`: uniform$(0, 5)$
3) Lexicon size	lexicon size ∼ context ∗ RSA + (1|dataset)	`Intercept`:uniform$(0, 1460)$, `sigma`: uniform$(0, 100)$
4) Lexicon informativeness	lexicon info ∼ context ∗ RSA + (1|dataset)	`Intercept`:uniform$(0, 9)$, `sigma`: uniform$(0, 2)$
5) Lexicon size-concept ratio	lexicon size-concept ratio ∼ context ∗ RSA + (1|dataset)	`Intercept`:uniform$(0, 1)$, `sigma`: uniform$(0, 0.1)$

Table C. Posterior summaries of the Bayesian hierarchical models predicting the entropy-based scores, i.e. NMI, effectiveness and consistency.

Model	Parameter	Median	95% CrI	pd	ROPE	% in ROPE	Rhat	ESS	Interpretation
NMI	condition	-0.02	[-0.03, 0.00]	99.50%	[-0.01, 0.01]	23.87%	1.000	3078	probably existing, of undecided significance
	fixed attributes	-0.04	[-0.04, -0.03]	100%	[-0.01, 0.01]	0%	1.000	6032	certainly existing, significant
	interaction	0.06	[0.05, 0.07]	100%	[-0.01, 0.01]	0%	1.000	3033	certainly existing, significant
effectiveness	condition	0.05	[0.03, 0.07]	100%	[-0.02, 0.02]	0%	1.001	2028	certainly existing, significant
	fixed attributes	-0.08	[-0.10, -0.07]	100%	[-0.02, 0.02]	0%	1.002	3040	certainly existing, significant
	interaction	0.07	[0.05, 0.08]	100%	[-0.02, 0.02]	0%	1.001	1949	certainly existing, significant
consistency	condition	-0.06	[-0.07, -0.05]	100%	[-0.01, 0.01]	0%	1.001	2307	certainly existing, significant
	fixed attributes	0.006	[0.00, 0.01]	98.88%	[-0.01, 0.01]	68.9%	0.999	3769	likely existing, of undecided significance
	interaction	0.04	[0.04, 0.05]	100%	[-0.01, 0.01]	0%	1.000	2402	certainly existing, significant

Mean entropy scores and standard deviations over five runs are calculated for each dataset and both conditions.
