# Supplementary figures and images for: The role of pragmatic mechanisms in referential communication and categorization: An emergent communication model
Source: PLoS Comput Biol. 2026 May 26;22(5):e1014326. doi: 10.1371/journal.pcbi.1014326 (PMC13252840; doi:10.1371/journal.pcbi.1014326)

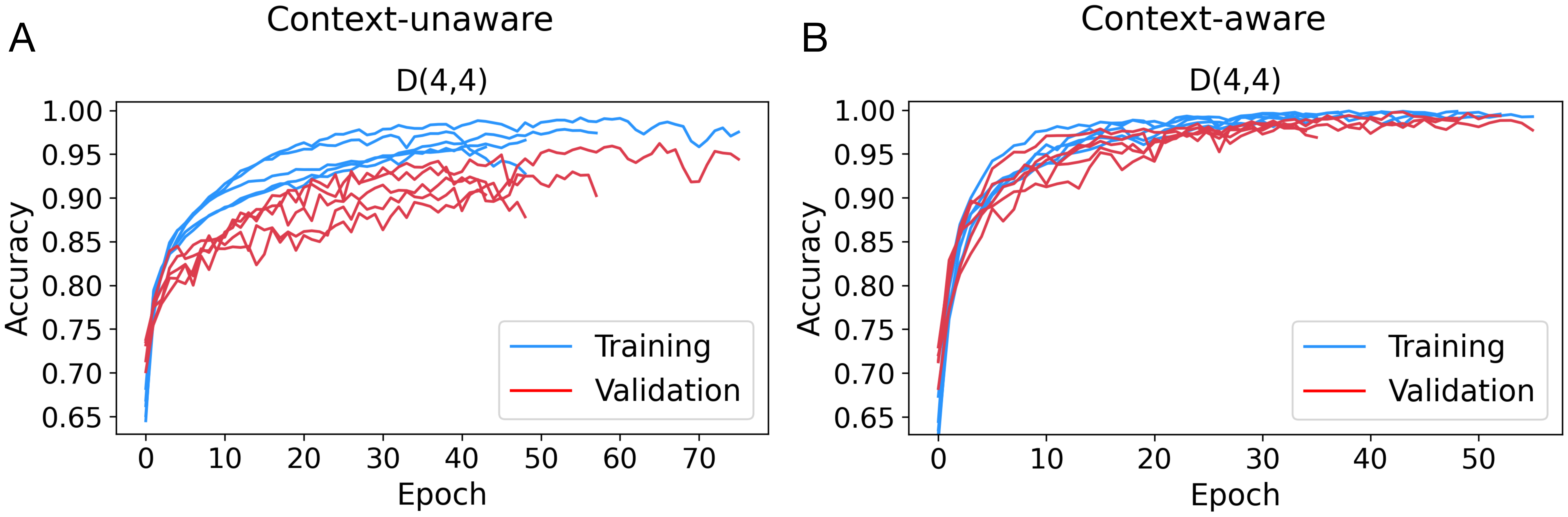

Supplement: S1 Fig — In S1 Fig, we show the training trajectories of five runs trained on the training and validation datasets of D(4,4) when training context-unaware (S1A Fig) and context-aware (S1B Fig) agents. (TIF) [file pcbi.1014326.s003.tif]

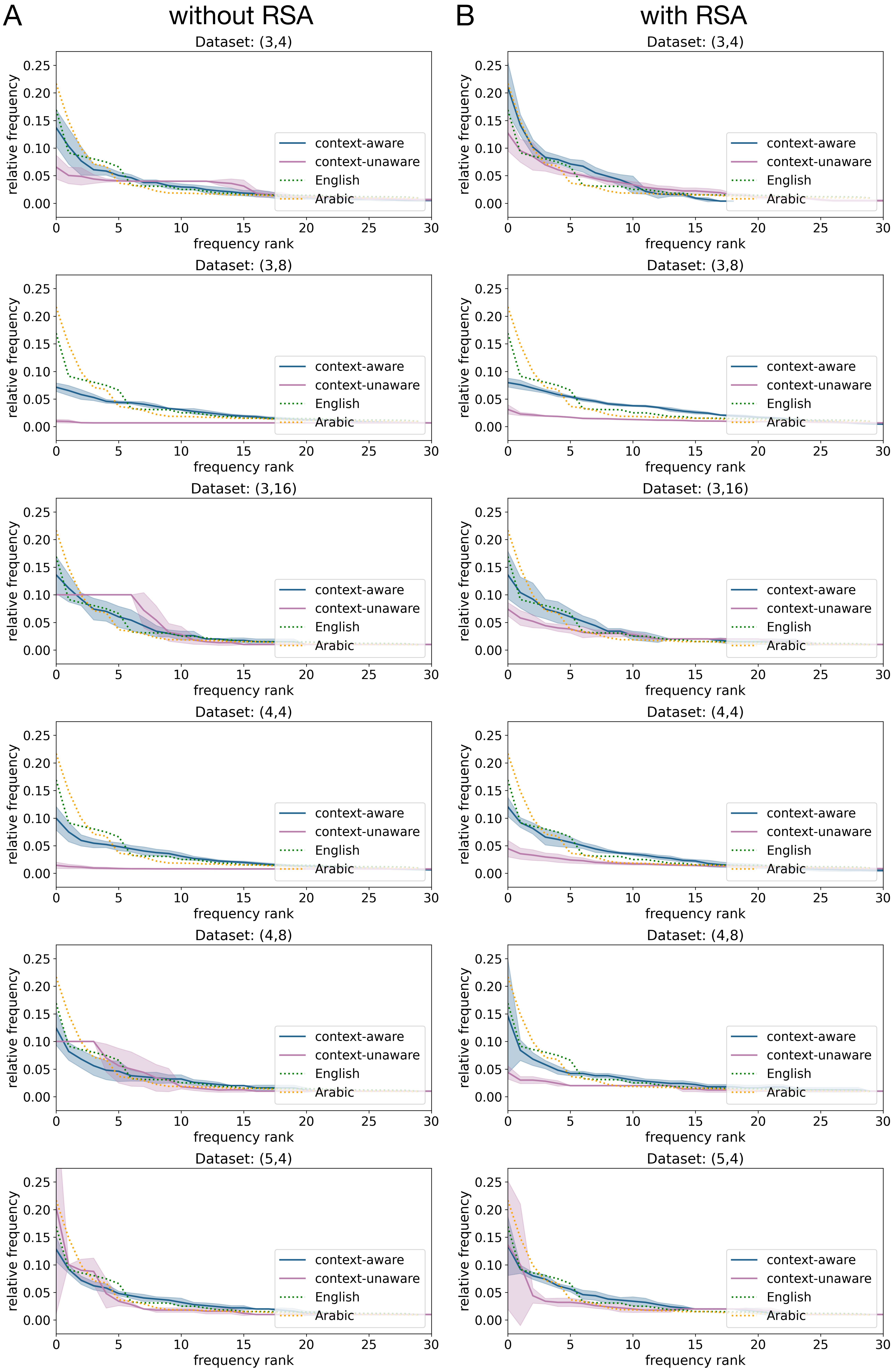

Supplement: S2 Fig — In S2 Fig, we present the frequency rank distributions for each dataset and for both context-unaware and context-aware trained agents without RSA (S2 FigA) and with RSA (S2 FigB). (TIF) [file pcbi.1014326.s005.tif]

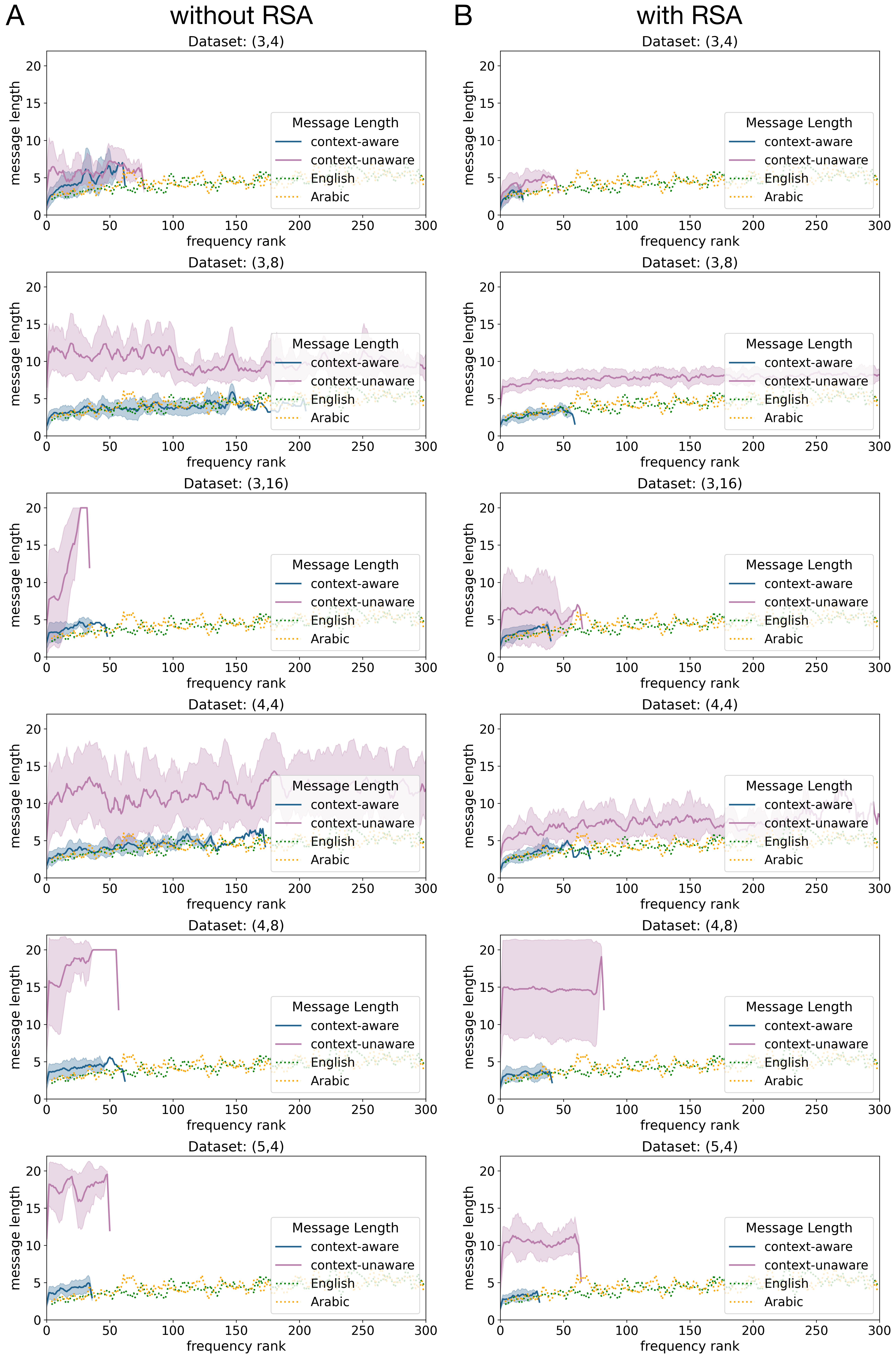

Supplement: S3 Fig — shows the message length frequency rank distributions for each dataset and for both context-unaware and context-aware trained agents without RSA (S3 FigA) and with RSA (S3 FigB). Curves are smoothed using a sliding average of five for better visibility. Arabic words have been stripped of diachritics with the library PyArabic [94] before calculating their length. (TIF) [file pcbi.1014326.s006.tif]
