# Supplementary material for: The role of pragmatic mechanisms in referential communication and categorization: An emergent communication model
Source: PLoS Comput Biol. 2026 May 26;22(5):e1014326. doi: 10.1371/journal.pcbi.1014326 (PMC13252840; doi:10.1371/journal.pcbi.1014326)
Supplement: S3 Appendix — This text identifies ambiguous trials in the test dataset and provides an RSA rationale. (PDF) [file pcbi.1014326.s007.pdf]

S3 Appendix

Amount of ambiguous trials in the test dataset

When adding utility-based pragmatic reasoning, we expected an improvement in efficiency of the language. To calibrate expectations of how much more efficient we can expect the languages to get by involving RSA, one of our reviewers suggested to estimate the amount of relevant test cases, following an argument made by Sikos et al. [1]. They analyzed the dataset of reference games used in the original RSA publication by Frank and Goodman [2] and found that only a small amount of trials are actually trials where the RSA pragmatic listener reasoning is needed.

Going back to our datasets, we analyzed the types of reference games included. The main difference to the standard RSA publications is, of course, the communication about target concepts comprised of multiple target objects. These are presented in different context conditions which are manipulated by a systematic deviation between target and distractor objects, whereby zero to n-1 attributes are shared between targets and distractors. Those reference games where distractors share 0 attributes with the targets are classified as unambiguous and deemed uninteresting for the RSA reasoning. However, reference games in which the distractors share at least one attribute with the target are ambiguous and relevant to the RSA reasoning investigated in this study. This is shown in Fig A, assuming that agents came up with languages that allow them to differentiate between the different attributes with different kinds of messages as we saw at least for the context-aware agents in the qualitative analysis (Table 6 in the main text).

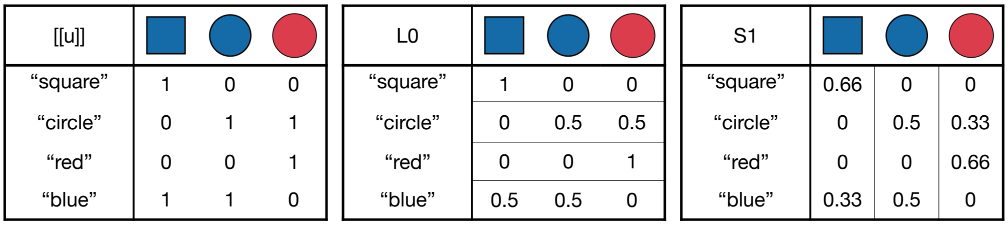

Fig A. RSA reasoning example. A simple reference game where the target concept blue square needs to be discriminated from distractor objects that partially share the same color.

Notably, the example shown is one of the examples deemed not relevant to RSA in [1]. However, this is due to the fact that they look at a level-1 pragmatic listener. We, on the other hand, look at a level-1 pragmatic speaker. Looking at the meaning matrix, two utterances, “square” and “blue” are available to refer to the blue square (or blue squares of different sizes in our concept game). It is only when a level-1 speaker reasons about a level-0 listener that the speaker can infer that the utterance “square” would in this context (where distractor objects share the color attribute but not the shape attribute with the target concept) be more informative than “blue”. Therefore, we argue that such ambiguous trials are relevant for the RSA speakers we model. Using the terminology introduced in [3], we model a Gricean level-1 speaker and look at *simple* reference games only. In such reference games, some of the messages available to describe the target are ambiguous. This is the case in our dataset for about two-thirds of the trials (see Table A).

Dataset	Total number of games	Ambiguous games	Ratio
D(3,4)	250	150	0.60
D(3,8)	1460	908	0.62
D(3,16)	100	56	0.56
D(4,4)	1250	799	0.64
D(4,8)	100	73	0.73
D(5,4)	100	76	0.76

Table A. Ratio of ambiguous games in the test datasets.

References

1. Sikos L, Venhuizen NJ, Drenhaus H, Crocker MW. Reevaluating pragmatic reasoning in language games. PLOS ONE. 2021;16(3):e0248388. doi:10.1371/journal.pone.0248388.

2. Frank MC, Goodman ND. Predicting pragmatic reasoning in language games. Science. 2012;336(6084):998. doi:10.1126/science.1218633.

3. Franke M, Degen J. Reasoning in Reference Games: Individual- vs. Population-Level Probabilistic Modeling. PLOS ONE. 2016;11(5):e0154854. doi:10.1371/journal.pone.0154854.
