# Supplementary material for: The role of pragmatic mechanisms in referential communication and categorization: An emergent communication model
Source: PLoS Comput Biol. 2026 May 26;22(5):e1014326. doi: 10.1371/journal.pcbi.1014326 (PMC13252840; doi:10.1371/journal.pcbi.1014326)
Supplement: Attachment — Submitted filename: PlosCB_ResponseLetter.pdf [file pcbi.1014326.s008.pdf]

Dear Editor,

We thank you and the reviewers for the constructive feedback on our manuscript titled “The role of pragmatic mechanisms in referential communication and categorization: An emergent communication model.” We have addressed all reviewer comments in our revision and have highlighted changes to the manuscript in yellow. Below you find our responses to reviewer comments and your own comments. Thank you once more for your help and prompt feedback.

Kind regards

LEGEND

black = author response
grey = reviewer comment

Additional Editor Comments:

The manuscript has been evaluated by two experts. They both agree that the paper makes an interesting contribution to the literature, and that it is -- in general -- suitable for publication in PLoS Computational Biology.
However, they also make a number of very constructive suggestions that I hope the authors will incorporate.
In particular, both referees mention that at least the gist of the methodology should already be mentioned in the main text (in addition to the Methods section). I fully support this view (even though I understand the authors followed the general journal structure). Having at least some informal summary in the main text (or an explicit reference to the Methods section) would be useful.

> Done - We have added an explicit reference to the Methods section in the main text and added more explicit information in the Introduction under Modeling approach as requested by the reviewers (see pages 4-6).

Reviewers' comments:

Reviewer's Responses to Questions

Comments to the Authors:
Please note here if the review is uploaded as an attachment.

Reviewer #1:

The paper explores how sensitivity to pragmatic considerations shapes the lexicon and categories in an emergent communication paradigm. The paper reports two experiments between artificial agents implemented as recurrent neural networks where the speaker agent tries to communicate a category to the listener, and the listener has to select the objects that qualify. Unlike standard reference games that use utterances to signal a particular object, this set up targets collections of objects, encouraging the emergence of categories (concepts).

I enjoyed reading this paper, and I think it makes an important contribution to our understanding of context-sensitivity. The approach the authors adopt is original, the experiments and the architecture are carefully documented.

I understand that having a methods section after the conclusions is the requirement of the journal. However, it makes it very difficult to appreciate some of the key details of the argument. For example, I've been wondering for a long time how informativeness is defined. In the Methods section, the authors explain that more informative descriptions mention more features. That makes a lot of sense. At the same time, the authors use the RSA-reasoning as one of the components of the architecture. In RSA, informativeness is not an absolute category but rather a relative one defined with respect to the alternative states and utterances in the model. The utterances are not necessarily hierarchical in standard RSA. I would find it helpful if at least informal definitions of the key concepts were integrated into the main text.

> Done - We added an explanation of our notion of informativeness in the section "Modeling approach" in the introduction (see page 5) and refer explicitly to the Methods section (on page 16) for the detailed description of the informativeness score we use in the evaluation (see pages 31-32).

Relatedly, the authors delay speaking about the pressure of the system to have shorter utterances until the methods section, which made me wonder how this behavior emerged in the model as I was reading through the results.

> Done - We added a short explanation of the length cost pressure in the section "Modeling approach" in the introduction: "On the speaker side, efficiency is achieved by a length cost pressure that incentivizes speakers to use shorter messages. On the listener side, informativeness is achieved by training the agents on communicative success." (page 4)

I generally followed the argument about context sensitivity and found it convincing. One piece of work that the authors might additionally mention is Brocchagen (2020) and his 2018 dissertation on signaling under uncertainty. There he develops an analytic model that predicts more ambiguity when context is shared. I feel like the current work makes an interesting contribution to this argument by exploring whether this pattern emerges in artificial language settings. Spelling out explicitly in which ways the current paper goes beyond analytic approaches (it certainly does) would help highlight its significance.

> Done - We added the references Brochhagen (2018) and Brochhagen (2020), and discussed their findings and how our approach goes beyond analytic approaches in the General discussion under Emergent communication and pragmatics (see page 23).

What remained not very clear for me is what including RSA components can show regarding the role of pragmatics. The authors explicitly state that one of the reasons why RSA models favor shorter descriptions in context-aware agents, is because RSA has a cost component, I appreciated this clarity! At the same time, I don't think there is enough information on how reasoning about utterance utility affects properties of the lexicon and/or categories. In order to demonstrate that RSA can at least in principle help with disambiguation, one would need to discuss in which scenarios pragmatic reasoning of the RSA-sort would be helpful. Sikos et al. (2021) have previously shown that in standard RSA reference games, many of the trials can be successfully completed without any pragmatic reasoning. I'm wondering if it were possible to identify cases where pragmatic reasoning about alternative utterances and states would make a difference in the current concept-level games? What would be even more relevant (and even harder) is to obtain an estimate of what proportion of trials would benefit from this style of reasoning. This information would give readers a way to calibrate expectations of how much can this reasoning in principle improve disambiguation given the settings of the games, and as a

consequence, how much more efficient would the lexicon/category system become when RSA is involved.

> Done - We added a footnote about the approximate amount of trials which we would classify as ambiguous trials where level-1 reasoning about the utility of their utterance should help the RSA speaker in our models to select the best utterance (see page 15): "Following a reviewer's suggestion, we estimated the amount of ambiguous games in our dataset where we would in principle expect level-1 RSA reasoning to help the speaker to select the best utterance. These make up about two thirds of the games in our test datasets (see S6 Appendix)." More future research is needed to draw definitive conclusions, but we hope that the identification of ambiguous trials will already help readers to calibrate their expectations on how much of a difference when involving RSA reasoning can in principle be expected in our setup.

I also didn't understand what context-unaware RSA is. In the RSA, as far as I know, reasoning about alternative utterances and states crucially depends on the objects present in the scene, and as a consequence, on the set of available utterances. Could you please clarify how the RSA-reasoning would unfold without context?

> Done - We have clarified that the way the RSA-reasoning is implemented for the speaker in Experiment 2 does not differ between the context-aware and context-unaware conditions in the section "Overview of experiments". In Experiment 2, we used the languages that emerged in the context-aware and context-unaware conditions from Experiment 1, but there no longer is a training phase. The difference between the RSA + context unaware and RSA + context aware conditions is whether the language used by the speaker agent emerged in the context-aware or the context-unaware condition in Experiment 1. Thus, what the comparison can tell us is how well RSA speakers who reason about their listeners' likely interpretation can leverage the languages that emerged with or without context for efficient communication (see page 9). To provide an intuition, the RSA + context-unaware condition can be thought of as a child with Theory of Mind abilities, but with no previous exposure to context. While arguably being artificial, this condition allows us to test a full 2x2 design crossing the role of context and the role of utility-based reasoning on efficient language use.

I struggle with the notion of synonymity that the authors adopt in the paper. I wouldn't call "dolmatian", "dog", and "animal" synonyms. They are synonyms in the game in a sense that all of these labels can refer to the same set of objects (I understand that these are just natural language examples for symbolic representations). I'm wondering if a quick definition of synonyms would be enough to clarify the confusion, or if a more neutral term would help (something like 'alternative descriptions'?).

> Done - We revised the use of the term "synonym" in the text and instead use "many-to-one mappings between messages and concepts" instead of "synonymous" and "one-to-many mappings between messages and concepts" instead of "polysemeous" expressions. We also clarified in the Methods section how these mappings relate to the conceptual hierarchy, namely that they can be alternative descriptions at different levels of the conceptual hierarchy (see page 33).

I found it very interesting that the authors make an argument not only about the development of the lexicon but also about the emergence of categories. However, I think some of these connections are not fully spelled out. The authors start by formulating the goals of the paper in terms of understanding how categorization works (p. 4): how categories affect emerging language and how language affects

categories. The latter is worked out less, I felt. The authors then proceed to formulate the main hypotheses which are now spelled out in terms of efficiency of language and the length of messages. So the hypotheses are spelled out in terms of linguistic outcomes. Right now I feel there is a mix of both language and categorization arguments, and I found at times, difficult to trace what the main focus is. It's possible that category/word distinction is sometimes difficult to differentiate given the set up: is that right that more complex concepts would have longer descriptions (at least in context-unaware settings)? This is not necessarily the case in natural languages: there can be single labels for very complex concepts with multiple properties. A clarification would be helpful!

Done - We revised the main questions in the introduction so that they lead more seamlessly toward the hypotheses we test. Specifically, we state that the focus is on how categories and communicative need are reflected in the emerging language and how the availability of pragmatic mechanisms influences this process. We have further clarified that our focus lies on the linguistic outcomes, i.e. how a language lexicalizes categories and a hierarchical category structure under the availability of different pragmatic mechanisms.

In response to the specific question raised above, we have clarified in the discussion that the effect that we find where more complex concepts have longer descriptions is due to our manipulation of communicative needs (see page 21): In principle, also in the emergent communication there could be single labels for very complex concepts. However, our setup uses compositional concepts which need to be discriminated based on single attribute features from related concepts on the same conceptual hierarchy. This leads to a communication strategy where it is useful to have specific symbols refer to specific attribute features because they can be combined and used flexibly in different messages to refer to different but related concepts. According to the communicative needs hypotheses, single labels for very complex concepts would be more likely to occur if the concept has to be communicated very often or if the information that is not included in the label can be frequently laid off on the context.

Overall, this is an impressive project with significant theoretical insights. I hope a clearer exposition would make these results appealing to a wider audience.

Minor comments:

Discussion sections (and a few other sections) contain very long paragraphs that span across whole pages. I would find it easier to follow the argument if those sections had more paragraphs each containing a separate idea.

Done - We have added more and revised the subheadings in the discussion as to separate arguments for language and categorization.

References

Brochhagen, T. (2020). Signalling under uncertainty: Interpretative alignment without a common prior. The British Journal for the Philosophy of Science.

Sikos L, Venhuizen NJ, Drenhaus H, Crocker MW (2021) Reevaluating pragmatic reasoning in language games. PLoS ONE 16(3): e0248388. https://doi.org/10.1371/journal.pone.0248388

Reviewer #2:

This paper presents and discusses two emergent communication experiments. The first experiment compares two conditions in which languages emerge for reference in a small blocks environment: one in which the context is taken into account by the speaker and one in which it isn't. Rather unsurprisingly, the one in which the context is taken in to account leads to more effective languages. The second experiment shows that linguistic efficiency improves in languages where agents use more pragmatic strategies.

My view on the main strengths of the work:

- The experiments were definitely non-trivial to set up.

- The experiments have been carried out rigorously.

For me personally, that suffices to warrant publication...

At the same time, I think a few things would need to be dealt with first:

- Literature: I was for example wondering why there are no references to a large body of work on multi-agent language emergence experiments where discriminative learning based on the environment was the driving factor, for example in concept emergence (but also grammar). This ranges form Steels' seminal talking heads experiment (https://langsci-press.org/catalog/book/49) to recent experiments such as Boktoko Ekila et al.'s recent AAMAS paper (https://arxiv.org/pdf/2401.08461). I think this body of work is at least worth mentioning (and in a paper of this length actually discussing).

> Done - We added these references and discuss them in the Introduction and General discussion (see pages 7 and 22-23).

- The authors take this particular emergent communciation framework as a given - that's what they do. That's fine - I understand, but is this really the best methodology to approach the research question (about the role of pragmatic mechanisms in referential communication)? Aren't the experiments more about emergent communication in artificial agent than about pragmatics in human languages? I'm not saying the experiments aren't adequate, but maybe the research question could be rephrased in a more focused way. I think this is mainly a contribution to the field of emergent communication, rather than to the field of pragmatics?

> Done - We rephrased the research question (page 3) and clarified in the general discussion what the emergent communication models, and our results more specifically, can add to the field of pragmatics under "Emergent communication as a framework to study pragmatics" (see pages 23-24): "First, we do not only investigate the role of context-based pragmatic mechanisms, but also the role of utility-based pragmatic mechanisms traditionally considered in the field of pragmatics, in the emergence of novel communication systems. Second, we put more focus on investigating the emerged languages in novel communicative interactions to see more directly which features of the emergent language are especially useful for (future) communication."

My view on the main limitations/shortcomings/weaknesses of the work:

- The paper is reasonably well-written, but for a paper of this length, I would have preferred to see more of the methods earlier on and integrated into the main flow of the text.

Done - We included more information on the methods in the Introduction under “Modeling approach” (see pages 4-6) and added an explicit reference to the Methods section on page 6 in the main text to address this point while also adhering to the journal standards which place the Methods after the Results section.

- I would have liked to see more focus on the experiments themselves and less 'general' discussion and conclusions. I'm sure the experiments contribute to this particular framework, but I would be more careful in drawing conclusions about human languages. I'm sure that the authors would agree that they only model tiny aspects of pragmatics. I haven't really learnt anything new about human language and communication - that's okay - but after reading the paper I also don't really clearly see what is to be concluded for emergent communication.

Done - We added a paragraph about our contributions to the emergent communication literature and a discussion of the question whether these models can inform our understanding of human language in the General discussion under “Emergent communication as a framework to study pragmatics” (see pages 23-24). In our view, pragmatics shapes communicative interactions repeatedly in the evolution of a language (be it between humans or agents). We employ the emergent communication models to model language evolution and in how far the availability of pragmatics shapes the evolutionary process as well as the use of the languages after they evolved. Our main finding that pragmatic mechanisms shape (the use of) an emerging or evolving language can be interpreted more generally for language systems that emerge between interacting agents (be it humans or artificial agents) under the pressures of efficiency and informativeness.

Have the authors made all data and (if applicable) computational code underlying the findings in their manuscript fully available?
The PLOS Data policy requires authors to make all data and code underlying the findings described in their manuscript fully available without restriction, with rare exception (please refer to the Data Availability Statement in the manuscript PDF file). The data and code should be provided as part of the manuscript or its supporting information, or deposited to a public repository. For example, in addition to summary statistics, the data points behind means, medians and variance measures should be available. If there are restrictions on publicly sharing data or code —e.g. participant privacy or use of data from a third party—those must be specified.

Reviewer #1: Yes

Reviewer #2: None

Done - We have added the link to the Github repository in the main text (page 10).
