# Supplementary material for: The role of pragmatic mechanisms in referential communication and categorization: An emergent communication model
Source: PLoS Comput Biol. 2026 May 26;22(5):e1014326. doi: 10.1371/journal.pcbi.1014326 (PMC13252840; doi:10.1371/journal.pcbi.1014326)
Supplement: Peer review history

# Peer review: Decision Letter 0

**DOI:** 10.1371/journal.pcbi.1014326.r001

**Authors:** Natalia L. Komarova, Christian Hilbe

6 Oct 2025

-->PCOMPBIOL-D-25-01198

The role of pragmatic mechanisms in referential communication and categorization: An emergent communication model

PLOS Computational Biology

Dear Dr. Kobrock,

Thank you for submitting your manuscript to PLOS Computational Biology. After careful consideration, we feel that it has merit but does not fully meet PLOS Computational Biology's publication criteria as it currently stands. Therefore, we invite you to submit a revised version of the manuscript that addresses the points raised during the review process.

Please submit your revised manuscript within 60 days Dec 06 2025 11:59PM. If you will need more time than this to complete your revisions, please reply to this message or contact the journal office at ploscompbiol@plos.org. When you're ready to submit your revision, log on to https://www.editorialmanager.com/pcompbiol/ and select the 'Submissions Needing Revision' folder to locate your manuscript file.

Please include the following items when submitting your revised manuscript:

* A rebuttal letter that responds to each point raised by the editor and reviewer(s). You should upload this letter as a separate file labeled 'Response to Reviewers'. This file does not need to include responses to formatting updates and technical items listed in the 'Journal Requirements' section below.

* A marked-up copy of your manuscript that highlights changes made to the original version. You should upload this as a separate file labeled 'Revised Manuscript with Track Changes'.

* An unmarked version of your revised paper without tracked changes. You should upload this as a separate file labeled 'Manuscript'.

If you would like to make changes to your financial disclosure, competing interests statement, or data availability statement, please make these updates within the submission form at the time of resubmission. Guidelines for resubmitting your figure files are available below the reviewer comments at the end of this letter

We look forward to receiving your revised manuscript.

Kind regards,

Christian Hilbe

Academic Editor

PLOS Computational Biology

Natalia Komarova

Section Editor

PLOS Computational Biology

**Additional Editor Comments:**

The manuscript has been evaluated by two experts. They both agree that the paper makes an interesting contribution to the literature, and that it is -- in general -- suitable for publication in PLoS Computational Biology.

However, they also make a number of very constructive suggestions that I hope the authors will incorporate.

In particular, both referees mention that at least the gist of the methodology should already be mentioned in the main text (in addition to the Methods section). I fully support this view (even though I understand the authors followed the general journal structure). Having at least some informal summary in the main text (or an explicit reference to the Methods section) would be useful.

**Journal Requirements:**

If the reviewer comments include a recommendation to cite specific previously published works, please review and evaluate these publications to determine whether they are relevant and should be cited. There is no requirement to cite these works unless the editor has indicated otherwise.

1) Please ensure that the CRediT author contributions listed for every co-author are completed accurately and in full.

At this stage, the following Authors/Authors require contributions: Kristina Kobrock, Elia Bruni, Nicole Gotzner, and Xenia Ohmer. Please ensure that the full contributions of each author are acknowledged in the "Add/Edit/Remove Authors" section of our submission form.

The list of CRediT author contributions may be found here: https://journals.plos.org/ploscompbiol/s/authorship#loc-author-contributions

2) We ask that a manuscript source file is provided at Revision. Please upload your manuscript file as a .doc, .docx, .rtf or .tex. If you are providing a .tex file, please upload it under the item type u2018LaTeX Source Fileu2019 and leave your .pdf version as the item type u2018Manuscriptu2019.

3) Some material included in your submission may be copyrighted. According to PLOSu2019s copyright policy, authors who use figures or other material (e.g., graphics, clipart, maps) from another author or copyright holder must demonstrate or obtain permission to publish this material under the Creative Commons Attribution 4.0 International (CC BY 4.0) License used by PLOS journals. Please closely review the details of PLOSu2019s copyright requirements here: PLOS Licenses and Copyright. If you need to request permissions from a copyright holder, you may use PLOS's Copyright Content Permission form.

Please respond directly to this email and provide any known details concerning your material's license terms and permissions required for reuse, even if you have not yet obtained copyright permissions or are unsure of your material's copyright compatibility. Once you have responded and addressed all other outstanding technical requirements, you may resubmit your manuscript within Editorial Manager.

Potential Copyright Issues:

i) Figures 1, 2, and 3. Please confirm whether you drew the images / clip-art within the figure panels by hand. If you did not draw the images, please provide (a) a link to the source of the images or icons and their license / terms of use; or (b) written permission from the copyright holder to publish the images or icons under our CC BY 4.0 license. Alternatively, you may replace the images with open source alternatives. See these open source resources you may use to replace images / clip-art:

- https://commons.wikimedia.org

- https://openclipart.org/.

4) Please amend your detailed Financial Disclosure statement. This is published with the article. It must therefore be completed in full sentences and contain the exact wording you wish to be published.

1) State what role the funders took in the study. If the funders had no role in your study, please state: "The funders had no role in study design, data collection and analysis, decision to publish, or preparation of the manuscript."

2) If any authors received a salary from any of your funders, please state which authors and which funders..

If you did not receive any funding for this study, please simply state: u201cThe authors received no specific funding for this work.u201d

1) Please ensure that the CRediT author contributions listed for every co-author are completed accurately and in full.

At this stage, the following Authors/Authors require contributions: Kristina Kobrock, Elia Bruni, Nicole Gotzner, and Xenia Ohmer. Please ensure that the full contributions of each author are acknowledged in the "Add/Edit/Remove Authors" section of our submission form.

The list of CRediT author contributions may be found here: https://journals.plos.org/ploscompbiol/s/authorship#loc-author-contributions

2) We ask that a manuscript source file is provided at Revision. Please upload your manuscript file as a .doc, .docx, .rtf or .tex. If you are providing a .tex file, please upload it under the item type u2018LaTeX Source Fileu2019 and leave your .pdf version as the item type u2018Manuscriptu2019.

3) Some material included in your submission may be copyrighted. According to PLOSu2019s copyright policy, authors who use figures or other material (e.g., graphics, clipart, maps) from another author or copyright holder must demonstrate or obtain permission to publish this material under the Creative Commons Attribution 4.0 International (CC BY 4.0) License used by PLOS journals. Please closely review the details of PLOSu2019s copyright requirements here: PLOS Licenses and Copyright. If you need to request permissions from a copyright holder, you may use PLOS's Copyright Content Permission form.

Please respond directly to this email and provide any known details concerning your material's license terms and permissions required for reuse, even if you have not yet obtained copyright permissions or are unsure of your material's copyright compatibility. Once you have responded and addressed all other outstanding technical requirements, you may resubmit your manuscript within Editorial Manager.

Potential Copyright Issues:

i) Figures 1, 2, and 3. Please confirm whether you drew the images / clip-art within the figure panels by hand. If you did not draw the images, please provide (a) a link to the source of the images or icons and their license / terms of use; or (b) written permission from the copyright holder to publish the images or icons under our CC BY 4.0 license. Alternatively, you may replace the images with open source alternatives. See these open source resources you may use to replace images / clip-art:

- https://commons.wikimedia.org

- https://openclipart.org/.

4) Please amend your detailed Financial Disclosure statement. This is published with the article. It must therefore be completed in full sentences and contain the exact wording you wish to be published.

1) State what role the funders took in the study. If the funders had no role in your study, please state: "The funders had no role in study design, data collection and analysis, decision to publish, or preparation of the manuscript."

2) If any authors received a salary from any of your funders, please state which authors and which funders..

If you did not receive any funding for this study, please simply state: u201cThe authors received no specific funding for this work.u201d

**Reviewers' comments:**

Reviewer's Responses to Questions

**Comments to the Authors:**

**Please note here if the review is uploaded as an attachment.**

Reviewer #1: The paper explores how sensitivity to pragmatic considerations shapes the lexicon and categories in an emergent communication paradigm. The paper reports two experiments between artificial agents implemented as recurrent neural networks where the speaker agent tries to communicate a category to the listener, and the listener has to select the objects that qualify. Unlike standard reference games that use utterances to signal a particular object, this set up targets collections of objects, encouraging the emergence of categories (concepts).

I enjoyed reading this paper, and I think it makes an important contribution to our understanding of context-sensitivity. The approach the authors adopt is original, the experiments and the architecture are carefully documented.

I understand that having a methods section after the conclusions is the requirement of the journal. However, it makes it very difficult to appreciate some of the key details of the argument. For example, I’ve been wondering for a long time how informativeness is defined. In the Methods section, the authors explain that more informative descriptions mention more features. That makes a lot of sense. At the same time, the authors use the RSA-reasoning as one of the components of the architecture. In RSA, informativeness is not an absolute category but rather a relative one defined with respect to the alternative states and utterances in the model. The utterances are not necessarily hierarchical in standard RSA. I would find it helpful if at least informal definitions of the key concepts were integrated into the main text.

Relatedly, the authors delay speaking about the pressure of the system to have shorter utterances until the methods section, which made me wonder how this behavior emerged in the model as I was reading through the results.

I generally followed the argument about context sensitivity and found it convincing. One piece of work that the authors might additionally mention is Brocchagen (2020) and his 2018 dissertation on signaling under uncertainty. There he develops an analytic model that predicts more ambiguity when context is shared. I feel like the current work makes an interesting contribution to this argument by exploring whether this pattern emerges in artificial language settings. Spelling out explicitly in which ways the current paper goes beyond analytic approaches (it certainly does) would help highlight its significance.

What remained not very clear for me is what including RSA components can show regarding the role of pragmatics. The authors explicitly state that one of the reasons why RSA models favor shorter descriptions in context-aware agents, is because RSA has a cost component, I appreciated this clarity! At the same time, I don’t think there is enough information on how reasoning about utterance utility affects properties of the lexicon and/or categories. In order to demonstrate that RSA can at least in principle help with disambiguation, one would need to discuss in which scenarios pragmatic reasoning of the RSA-sort would be helpful. Sikos et al. (2021) have previously shown that in standard RSA reference games, many of the trials can be successfully completed without any pragmatic reasoning. I’m wondering if it were possible to identify cases where pragmatic reasoning about alternative utterances and states would make a difference in the current concept-level games? What would be even more relevant (and even harder) is to obtain an estimate of what proportion of trials would benefit from this style of reasoning. This information would give readers a way to calibrate expectations of how much can this reasoning in principle improve disambiguation given the settings of the games, and as a consequence, how much more efficient would the lexicon/category system become when RSA is involved.

I also didn’t understand what context-unaware RSA is. In the RSA, as far as I know, reasoning about alternative utterances and states crucially depends on the objects present in the scene, and as a consequence, on the set of available utterances. Could you please clarify how the RSA-reasoning would unfold without context?

I struggle with the notion of synonymity that the authors adopt in the paper. I wouldn’t call “dolmatian”, “dog”, and “animal” synonyms. They are synonyms in the game in a sense that all of these labels can refer to the same set of objects (I understand that these are just natural language examples for symbolic representations). I’m wondering if a quick definition of synonyms would be enough to clarify the confusion, or if a more neutral term would help (something like ‘alternative descriptions’?).

I found it very interesting that the authors make an argument not only about the development of the lexicon but also about the emergence of categories. However, I think some of these connections are not fully spelled out. The authors start by formulating the goals of the paper in terms of understanding how categorization works (p. 4): how categories affect emerging language and how language affects categories. The latter is worked out less, I felt. The authors then proceed to formulate the main hypotheses which are now spelled out in terms of efficiency of language and the length of messages. So the hypotheses are spelled out in terms of linguistic outcomes. Right now I feel there is a mix of both language and categorization arguments, and I found at times, difficult to trace what the main focus is. It’s possible that category/word distinction is sometimes difficult to differentiate given the set up: is that right that more complex concepts would have longer descriptions (at least in context-unaware settings)? This is not necessarily the case in natural languages: there can be single labels for very complex concepts with multiple properties. A clarification would be helpful!

Overall, this is an impressive project with significant theoretical insights. I hope a clearer exposition would make these results appealing to a wider audience.

Minor comments:

Discussion sections (and a few other sections) contain very long paragraphs that span across whole pages. I would find it easier to follow the argument if those sections had more paragraphs each containing a separate idea.

References

Brochhagen, T. (2020). Signalling under uncertainty: Interpretative alignment without a common prior. The British Journal for the Philosophy of Science.

Sikos L, Venhuizen NJ, Drenhaus H, Crocker MW (2021) Reevaluating pragmatic reasoning in language games. PLoS ONE 16(3): e0248388. https://doi.org/10.1371/journal.pone.0248388

Reviewer #2: This paper presents and discusses two emergent communication experiments. The first experiment compares two conditions in which languages emerge for reference in a small blocks environment: one in which the context is taken into account by the speaker and one in which it isn't. Rather unsurprisingly, the one in which the context is taken in to account leads to more effective languages. The second experiment shows that linguistic efficiency improves in languages where agents use more pragmatic strategies.

My view on the main strengths of the work:

- The experiments were definitely non-trivial to set up.

- The experiments have been carried out rigorously.

For me personally, that suffices to warrant publication...

At the same time, I think a few things would need to be dealt with first:

- Literature: I was for example wondering why there are no references to a large body of work on multi-agent language emergence experiments where discriminative learning based on the environment was the driving factor, for example in concept emergence (but also grammar). This ranges form Steels' seminal talking heads experiment (https://langsci-press.org/catalog/book/49) to recent experiments such as Boktoko Ekila et al.'s recent AAMAS paper (https://arxiv.org/pdf/2401.08461). I think this body of work is at least worth mentioning (and in a paper of this length actually discussing).

- The authors take this particular emergent communciation framework as a given - that's what they do. That's fine - I understand, but is this really the best methodology to approach the research question (about the role of pragmatic mechanisms in referential communication)? Aren't the experiments more about emergent communication in artificial agent than about pragmatics in human languages? I'm not saying the experiments aren't adequate, but maybe the research question could be rephrased in a more focused way. I think this is mainly a contribution to the field of emergent communication, rather than to the field of pragmatics?

My view on the main limitations/shortcomings/weaknesses of the work:

- The paper is reasonably well-written, but for a paper of this length, I would have preferred to see more of the methods earlier on and integrated into the main flow of the text.

- I would have liked to see more focus on the experiments themselves and less 'general' discussion and conclusions. I'm sure the experiments contribute to this particular framework, but I would be more careful in drawing conclusions about human languages. I'm sure that the authors would agree that they only model tiny aspects of pragmatics. I haven't really learnt anything new about human language and communication - that's okay - but after reading the paper I also don't really clearly see what is to be concluded for emergent communication.

**********

**Have the authors made all data and (if applicable) computational code underlying the findings in their manuscript fully available?**

The PLOS Data policy requires authors to make all data and code underlying the findings described in their manuscript fully available without restriction, with rare exception (please refer to the Data Availability Statement in the manuscript PDF file). The data and code should be provided as part of the manuscript or its supporting information, or deposited to a public repository. For example, in addition to summary statistics, the data points behind means, medians and variance measures should be available. If there are restrictions on publicly sharing data or code —e.g. participant privacy or use of data from a third party—those must be specified.

Reviewer #1: Yes

Reviewer #2: None

**********

PLOS authors have the option to publish the peer review history of their article (what does this mean?). If published, this will include your full peer review and any attached files.

If you choose “no”, your identity will remain anonymous but your review may still be made public.

**Do you want your identity to be public for this peer review?**  For information about this choice, including consent withdrawal, please see our Privacy Policy.

Reviewer #1: No

Reviewer #2: No

**Figure resubmission:**

While revising your submission, we strongly recommend that you use PLOS’s NAAS tool (https://ngplosjournals.pagemajik.ai/artanalysis) to test your figure files. NAAS can convert your figure files to the TIFF file type and meet basic requirements (such as print size, resolution), or provide you with a report on issues that do not meet our requirements and that NAAS cannot fix.-->

After uploading your figures to PLOS’s NAAS tool - https://ngplosjournals.pagemajik.ai/artanalysis, NAAS will process the files provided and display the results in the "Uploaded Files" section of the page as the processing is complete. If the uploaded figures meet our requirements (or NAAS is able to fix the files to meet our requirements), the figure will be marked as "fixed" above. If NAAS is unable to fix the files, a red "failed" label will appear above. When NAAS has confirmed that the figure files meet our requirements, please download the file via the download option, and include these NAAS processed figure files when submitting your revised manuscript.

**Reproducibility:**

To enhance the reproducibility of your results, we recommend that authors of applicable studies deposit laboratory protocols in protocols.io, where a protocol can be assigned its own identifier (DOI) such that it can be cited independently in the future. Additionally, PLOS ONE offers an option to publish peer-reviewed clinical study protocols. Read more information on sharing protocols at https://plos.org/protocols?utm_medium=editorial-email&utm_source=authorletters&utm_campaign=protocols

# Author comment: Author response to Decision Letter 1

**DOI:** 10.1371/journal.pcbi.1014326.r002

9 Apr 2026

Attachment Submitted filename: PlosCB_ResponseLetter.pdf

# Peer review: Decision Letter 1

**DOI:** 10.1371/journal.pcbi.1014326.r003

**Authors:** Natalia L. Komarova, Christian Hilbe

12 May 2026

Dear M.Sc. Kobrock,

We are pleased to inform you that your manuscript 'The role of pragmatic mechanisms in referential communication and categorization: An emergent communication model' has been provisionally accepted for publication in PLOS Computational Biology.

Before your manuscript can be formally accepted you will need to complete some formatting changes, which you will receive in a follow up email. A member of our team will be in touch with a set of requests.

Please note that your manuscript will not be scheduled for publication until you have made the required changes, so a swift response is appreciated.

IMPORTANT: The editorial review process is now complete. PLOS will only permit corrections to spelling, formatting or significant scientific errors from this point onwards. Requests for major changes, or any which affect the scientific understanding of your work, will cause delays to the publication date of your manuscript.

Should you, your institution's press office or the journal office choose to press release your paper, you will automatically be opted out of early publication. We ask that you notify us now if you or your institution is planning to press release the article. All press must be co-ordinated with PLOS.

Thank you again for supporting Open Access publishing; we are looking forward to publishing your work in PLOS Computational Biology.

Best regards,

Christian Hilbe

Academic Editor

PLOS Computational Biology

Natalia Komarova

Section Editor

PLOS Computational Biology

***********************************************************

Originally, the two referees suggested 'Major Revision' and 'Minor Revision', respectively. We have now sent the revised version to the referee who originally suggested a 'Major Revision', and this referee now suggests 'Acceptance'.

Overall, the authors have carefully revised their manuscript, so I concur with the reviewer.

Reviewer's Responses to Questions

**Comments to the Authors:**

**Please note here if the review is uploaded as an attachment.**

Reviewer #1: I would like to thank the authors for a thorough and careful revision of the manuscript! All of my concerns have been taken into account. I recommend the manuscript for publication.

**********

**Have the authors made all data and (if applicable) computational code underlying the findings in their manuscript fully available?**

The PLOS Data policy requires authors to make all data and code underlying the findings described in their manuscript fully available without restriction, with rare exception (please refer to the Data Availability Statement in the manuscript PDF file). The data and code should be provided as part of the manuscript or its supporting information, or deposited to a public repository. For example, in addition to summary statistics, the data points behind means, medians and variance measures should be available. If there are restrictions on publicly sharing data or code —e.g. participant privacy or use of data from a third party—those must be specified.

Reviewer #1: Yes

**********

PLOS authors have the option to publish the peer review history of their article (what does this mean?). If published, this will include your full peer review and any attached files.

If you choose “no”, your identity will remain anonymous but your review may still be made public.

**Do you want your identity to be public for this peer review?**  For information about this choice, including consent withdrawal, please see our Privacy Policy.

Reviewer #1: **Yes:**  Asya Achimova

# Editor report: Acceptance letter

**DOI:** 10.1371/journal.pcbi.1014326.r004

**Authors:** Natalia L. Komarova, Christian Hilbe

PCOMPBIOL-D-25-01198R1

The role of pragmatic mechanisms in referential communication and categorization: An emergent communication model

Dear Dr Kobrock,

I am pleased to inform you that your manuscript has been formally accepted for publication in PLOS Computational Biology. Your manuscript is now with our production department and you will be notified of the publication date in due course.

The corresponding author will soon be receiving a typeset proof for review, to ensure errors have not been introduced during production. Please review the PDF proof of your manuscript carefully, as this is the last chance to correct any errors. Please note that major changes, or those which affect the scientific understanding of the work, will likely cause delays to the publication date of your manuscript.

Soon after your final files are uploaded, unless you have opted out, the early version of your manuscript will be published online. The date of the early version will be your article's publication date. The final article will be published to the same URL, and all versions of the paper will be accessible to readers.

For Research, Software, and Methods articles, you will receive an invoice from PLOS for your publication fee after your manuscript has reached the completed accept phase. If you receive an email requesting payment before acceptance or for any other service, this may be a phishing scheme. Learn how to identify phishing emails and protect your accounts at https://explore.plos.org/phishing.

Thank you again for supporting PLOS Computational Biology and open-access publishing. We are looking forward to publishing your work!

With kind regards,

Anita Estes

PLOS Computational Biology | Carlyle House, Carlyle Road, Cambridge CB4 3DN | United Kingdom ploscompbiol@plos.org | Phone +44 (0) 1223-442824 | ploscompbiol.org | @PLOSCompBiol
